# PROCare4Life integrated care model validation

**DOI:** 10.12688/openreseurope.16678.3

**Published:** 2026-04-16

**Authors:** Pilar Gangas, Niamh Lennox-Chhugani, Arturo Alvarez, Edelweiss Aldasoro, Yusuf Can Semerci, Elda Judica, Claudia Louro, Joao P Proença, Mayca Marin, Elisa Calarota, Sarah Meidlinger

**Affiliations:** 1 International Foundation for Integrated Care (IFIC), The Base B Evert van de Beekstraat 1-104 Schiphol Airport, 1118 CBL, The Netherlands; 2 Maastricht University, Minderbroedersberg 4-6, Maastricht, 6211 LK, The Netherlands; 3 Department of Neurorehabilitation Sciences, Casa di Cura Igea, Milan, 20144, Italy; 4 Kinetikos, Campo Grande 28, 10º D, Lisbon, 1700-093, Portugal; 5 Asociacion Parkinson Madrid, Calle Andres Torrejon, 18, Madrid, 28014, Spain; 6 Wohlfahrtswerk für Baden-Württemberg, Schloßstraße 80, Stuttgart, 70176, Germany

**Keywords:** Integrated care; Evaluation; Validation; Assessment; Methodology; eHealth; Digital health; Parkinson’s; Alzheimer’s; Elderly; Dementia.

## Abstract

This article focuses on reporting the methodology and results of the EU funded project PROCare4Life, whose main goal has been to develop an integrated, personalised, IT system, to empower and improve the Quality of Life of elderly people living with Parkinsons’, Alzheimer’s or another dementia. Research has been implemented to evaluate the results against the PROCare4Life integrated care strategy. Multimethod, quantitative, and qualitative research has been performed, benefiting from the Pilot 3 results gathered by consortium members. The article has been organised according to the research questions, which were based on the expected results by PROCare4Life that might support the future integration of care when the system is further developed and implemented into healthcare provision organisations. The PROCare4Life system is a digital tool that includes features that might support the advance of the integration of care, such as data sharing among multidisciplinary healthcare professionals and with patients and caregivers, the enhancement of direct communication among them or the constant sharing of the data monitored by the PROCare4Life system. Technology readiness levels (TRLs) 7 was achieved by the final prototype of the PROCare4Life system. Pilot representatives consider the PROCare4Life idea and underlying model of care very innovative and promising and believe it can certainly improve care practice through more enhanced integrated care. It is usable for people of different ages, conditions, and socioeconomic levels. It has been assessed to be more effective for elderly people living with Parkinson’s than with dementia. Patients and caregivers indicated that the system helped them reduce their anxiety and increase their quality of life. Although overall assessment of the system by healthcare professionals was positive, divided opinions were shared on its capability to save time. Requirements for future scalability have been included in the last section.

## Introduction

PeRsOnalised Integrated CARE Solution for Elderly facing several short- or long-term conditions and enabling a better quality of Life (PROCare4Life) was an EU-funded project, implemented over the period of January 2020 through June 2023. According to the latest report by Alzheimer Europe, it is estimated that the number of people living with dementia in the EU was more than 7.8 million in 2018, which is expected to almost double by 2025, increasing to more than 14 million people. ^1^ Most dementias, around 60-70 per cent of the cases, correspond to Alzheimer’s. ^2^ Parkinson’s is a progressive, complex and neurodegenerative disease whose prevalence has been estimated in 1.2 million EU citizens, and is also estimated to double in the next 20 years. ^3^ These two conditions might involve cognitive and mobility reduced capabilities, that often require long term treatment and increased costs. The estimated combined cost of both conditions is 357 billion euro, ^4^ and its symptoms affect the quality of life of people living with any of those conditions. A main goal of PROCare4Life was to develop personalised technologies to support people living with dementia, Parkinson’s, their carers, and healthcare professionals. In total, 14 partners located in six EU counties gathered for three and a half years to create, from a multidisciplinary perspective, the PROCare4Life solution. Over the duration of the implementation of PROCare4Life, three waves of pilots in six pilot sites helped to codesign, fine-tune and improve the PROCare4Life system. The pilot sites were the following: Spitalul Universitar de Urgenta Bucuresti (Bucharest); Asociación Parkinson Madrid (Madrid); Casa di Cura Igea (Milan); Campus Neurológico Senior (Lisbon); University of Medicine and Pharmacy (Bucharest); and Wohlfahrtswerk für Baden-Württemberg (Stuttgart). These end users’ organisations have worked closely together with the technical partners developing the solution: Kinetikos (coordinators of PROCare4Life); Maastricht University (designing and developing the sensorial ecosystem); Universidad Politécnica Madrid (development of high-level subsystems); and Software Imagination & Vision S.R.L. (generation of social and communication services), Atos (design of the integrated care platform). Other partners were the University of Münster (advising on the social sciences methodology of the users’ needs and requirements identification and generating physical activity recommendations as well as training materials for the users), International Foundation of Integrated Care (focusing on the validation of the integrated care approach and dissemination and communication) as well as Stelar (ethical and legal issues).

**Figure 1.**
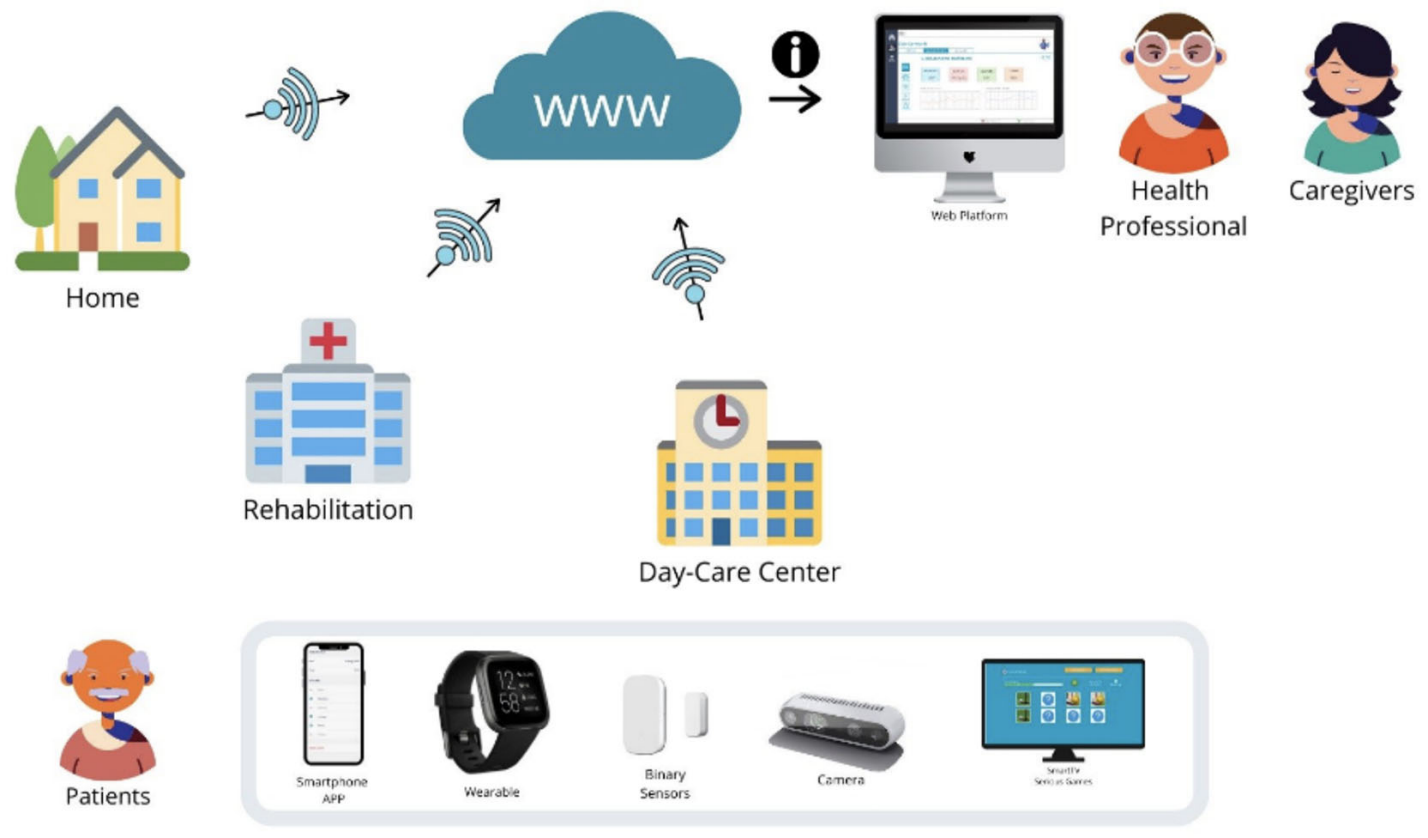
PROCare4Life users, scenarios, devices.

**Figure 2.**
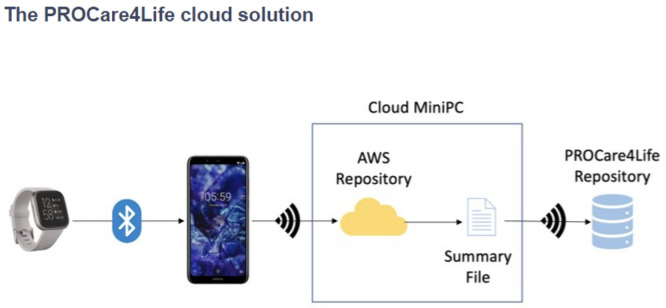
PROCare4Life Cloud Solution.

A combination of PROCare4Life technologies was deployed in three different scenarios: at the home of the people living with Parkinson’s, Dementia, or comorbidities; at rehabilitation rooms for improving physical capabilities; and at day care centres. The PROCare4Life general concept is summarised in Figure 1, while the cloud solution used in pilot 3 is depicted in Figure 2. Patients, caregivers, and healthcare professionals were involved in the care ecosystem that PROCare4Life supported. Data transfer was based in the Cloud gathering data from smartwatches and smartphones, including binary sensors at some of the home scenarios. In-depth cameras were incorporated in the rehabilitation rooms, since they were needed for the patient to be identified by the system following rehabilitation, using their personal body measures. Cognitive games were tested in both the home scenario and the rehabilitation one. Data coming from these different sources were combined and analysed for each individual, allowing for personalised content and recommendations in the phone app for patients and web app for healthcare professionals. ^5^

### PROCare4Life integrated care model

All EU countries have implemented integrated care initiatives, although their features, scope and extent have been considerably varied. ^6^ When addressing the importance of integration of care, the guidelines of the World Health Organisation (WHO) are a key consideration. The Declaration of Astana (2018) acknowledges that “fragmented approaches focusing on single diseases do not deliver on the goal of improving health for all”, based on real life experiences that have proven that “comprehensive, integrated, people-centred primary health care is feasible and achievable”. Among the lessons learned, several key points have been highlighted, some of them well connected with PROCare4Life system codesign process: contextualise and tailor the model of care to the pilot sites; strengthen multidisciplinary approaches using the technology; scale up capacity to identify and reach vulnerable people. ^7^

The PROCare4Life concept is an integrated care solution, which has developed personalised artificial intelligence (AI) algorithms to support better self-management, increase quality of life and empowerment of citizens living with Parkinson’s, dementia, or comorbidities. PROCare4Life has developed tailored functionalities to achieve these means, such as detection of symptoms and signs, detection of deviations or abnormal behaviour, data sharing and enhanced communication among the care ecosystem of each patient. Health and social data gathered were used to create an individual profile for each user, allowing for the PROCare4Life system to identify when their data or behaviour may deviate from their usual parameters. Training materials and personalised recommendations were provided to patients and caregivers, together with summaries of their healthcare values for their healthcare professionals, also reporting the main health events that were identified from both the usual and unusual data and behaviour of end users. All the pilot organisations have contributed significatively to PROCare4Life development, iterative testing and to the implementation of the PROCare4life model validation research, which is the focus of this article. The research process has been developed under the leadership of IFIC, who conceptualised the methodology and managed the internal fieldwork implementation.

The PROCare4Life system has been codesigned with 5,127 users in several waves, incorporating the needs and wishes of its future users iteratively: elderly people living with dementia or Parkinson’s, their carers, and their healthcare professionals. The system was also tested for elderly people living with comorbidities. It has been shown that information systems that are developed considering the real needs of the people that will be using them are more likely to have a successful adoption. ^8^ Particularly interesting is the personalization that AI provides when implementing technology enabled integrated person-centred care. ^9^ The EU is supporting the use of digital technologies for healthy ageing with initiatives such as the European Innovation Partnership on Active and Healthy Ageing (EIP on AHA) ^10^, or the project that has followed this network, the Innovation Networks for Scaling Active and Healthy Ageing (in4aha). ^11^ One of its main challenges is the scalability and transferability of good practices in Europe. ^12^ A means to improve the success of new integrated care models has been shown to codesign the digital solutions to make it truly people centred. ^13^ The codesign approach for developing integrated care technologies has been implemented in PROCare4Life and other EU funded projects, such as ValueCare, ^14^ VIGOUR, ^15^ INCAREHEART ^16^, CareMatrix, ^17^ eCare, ^18^ ROSIA ^19^, VCARE ^20^, IDEAFAST ^21^ or TeNDER ^22^.

Integrated care using digital tools requires that the technology is people-centred. ^23^ Among PROCare4Life features, participants highlighted its capability to gather health data constantly and the direct communication channels that the system provides for users to connect among themselves. Some key challenges to implementing new care models have been identified through participatory research, highlighting poor communications as one of the key barriers to the implementation of new care models ^24^, one of the aspects that PROCare4Life has addressed. Data sharing of the clinical and social care records, also part of PROCare4Life features, appear to be key components for the integration of care using digital technologies, despite the slow progress of digitalisation. ^25^ Both carers and patients have reported their anxiety to have been reduced due to using the PROCare4Life system, and their quality of life increased. Data sharing and direct communication with healthcare professionals have been highly appreciated by participants. These features are thus able to provide a good basis for advancing towards people-centred care and for multidisciplinary communication among the different profiles of healthcare professionals involved in providing care to each citizen, for an increased continuity and coordination in healthcare provision for elderly people living with Parkinson’s or dementia. ^26^ Understanding how technology can be designed to reduce the burden for healthcare professionals is another key component of digital integrated care models ^27^, and that has been also one of the objectives of PROCare4Life. To operationalise it, the opinions of the healthcare professionals focused on their capability to save their time.

PROCare4Life has implemented its large-scale pilots in six different pilot sites, not fully integrating the PROCare4Life technology into their current care pathways. The contexts ^28^ of the integrated care models is important to be considered, with the PROCare4Life system developing a flexible approach for each pilot site, acknowledging that the organisations that have delivered them are different among themselves. When addressing the opportunities provided by digital health tools, the analysis can be performed from several perspectives, such as clinical, professional, organizational or system level. ^29^ PROCare4Life pilots have tested the PROCare4Life technology in real-life environments. The integration types (linkage, coordination, and full integration), ^30^ of all the pilot sites have been identified to be implemented at the initial stages of integrated care linkage, involving sharing information across organizational and service previous boundaries. It can thus be concluded that the journey to further integrating care has been initiated using the PROCare4Life solution, although there is still a long way for its future full integration as addressed with more detail on the discussion section. ^31^

## Methods

An operative definition of integrated care includes three components: people centred, increased coordination, and continuity. These are the core dimensions used to develop the validation methodology for PROCare4Life. ^32^ Our evaluation needed to be adjusted to the actual implementation of the PROCare4Life prototype across the six pilots. As an innovation action, the expected level of development of the technology (TLR-7) did not forecast the full integration of PROCare4Life in the respective organisations implementing the pilots, but its iterative testing in real life scenarios was successfully implemented.

Gender has been considered in the design, sample selection, and data analysis strategies, making sure to differentiate between sex and gender. Appropriate numbers of participants, both males and females, were carefully monitored when selected, assuring an adequate balance of both genders. Other self-perceived gender identification was also monitored, to acknowledge the weight of other options, such as non-binary. 54% of the participants responded as males. When disaggregated by condition and gender, it was confirmed that 68% of participants living with dementia declared themselves as women (61% the average of women living with dementia ^33^). Among participants living with Parkinson’s, a higher proportion of men was included, 62,1%, also aligned with the higher percentage of males among people living with Parkinson’s, estimated in 63,12 per cent. ^34^ When analysing data, the differences in results by both genders have been explored, reporting by gender when relevant, particularly in the section focusing on equity.

The PROCare4Life consortium researched the effects of using PROCare4Life on several subjective aspects, such as usability or acceptance, combined with technical and clinical evaluations, creating a baseline through an entry interview that was repeated when leaving the study in an exit interview. PROCare4Life implemented three pilot waves, whose respective main goals and the number of participants are described below.

**Pilot 1.** Technical feasibility and usability of the PROCARE4LIFE system: 479 participants

**Pilot 2.** Characterization and validation of PROCARE4LIFE system metrics: 249 participants

**Pilot 3.** Validation and impact of the final version of PROCARE4LIFE system: 1,624

**Replicability study:** +100 people living with comorbidities. The replicability study researched the potential uses of PROCare4Life for other profiles of people; older adults living with comorbidities.

The ethical principles and GDPR regulations were followed in accordance with the general management guidelines established by the Kinetikos project coordination team, the Stellar ethical and legal committee leaders. Additionally, all studies were approved by the ethical committee in each country, and all the recommendations of the respective ethical committees implemented.

• **CNS (Portugal):** Campus Neurológico Sénior is a private healthcare institution specialized in neurological diseases, mainly in the fields of Parkinson’s disease, stroke, and Alzheimer’s disease.
• **CCI (Italy):** Casa di Cura IGEA is a multispecialty clinical centre that provides inpatient and outpatient services to neurological patients.
• **UMF/CCI (Romania):** UMF is the acronym for the Carol Davila University of Medicine and Pharmacy in Bucharest. Colentina Clinical Hospital (CCI) is a teaching hospital affiliated with UMF that offers inpatient and outpatient medical services for acute and chronic diseases, reimbursed by the Romanian national healthcare system.
• **UHB (Romania):** Spitalul Universitar de Urgență Bucureşti is the largest hospital in Bucharest under the jurisdiction of the Romanian Ministry of Health. The Neurology department includes a rehabilitation centre and a Neurology Clinic.
• **APM (Spain):** Asociación Parkinson Madrid is a non-profit organisation, based in Madrid composed of nearly 2,000 members and volunteers that provide care, information, counselling, training, etc., to people with Parkinson’s disease and their families.
• **WBW (Germany):** Wohlfahrtswerk für Baden-Württemberg is one of the biggest providers of residential services for older adults in Baden-Württemberg, from sheltered housing to in-house residential care, residential communities, and day care centres.

The aim of this article is strictly linked to reporting PROCare4Life integrated care model validation, through the pilots and phases of PROCare4Life implementation presented as contextual key factors to frame our analysis, although highlighting that some of the pilot 3 results have been used to form our evaluation. Pilot 3 focused on assessing the quality of life, cost effectiveness and impact on the six pilot sites, also including a control sample at each site, as all of these dimensions of analysis are relevant to this article. Pilot 3 was the most extensive codesign phase and provided the highest amount of quality data, led by Casa di Cura Egea.

### PROCare4Life integrated care model validation research questions, hypothesis, and key assumptions

Comparative longitudinal research has been implemented for the PROCare4Life integrated care model validation, including comparison by condition and scenario. The research questions, derived from the PROCare4Life Description of Action (DoA) are the following:

1. How has the PROCare4Life solution ‘fitted’ into the existing care pathway in the pilot sites?
2. What has been the effect of PROCare4Life on care performance, including:
  a. People living with dementia or Parkinson’s experience of care?
  b. Equity of access?
  c. People living with dementia or Parkinson’s quality of life?
  d. Frequency of adverse events/avoidable admissions to hospital?
  e. Caregivers’ quality of life?
3. What is the healthcare professionals’ efficiency?
4. How could the use of the PROCare4Life solution eventually make care pathways more integrated?
5. What conditions need to be in place across the system for the model to be more integrated?

Hypotheses were based on the expectation that the utilization of the PROCare4Life solution will:

1. Result in earlier intervention and management of common symptoms of PD and AD, which will:
  a. Reduce adverse events.
  b. Reduce avoidable admissions to the hospital.
  c. Reduce people living with dementia or Parkinson’s and caregiver anxiety.
  d. Improve quality of life.
  e. Improve workforce efficiency.
2. Enable streamlined multi-disciplinary communication across the pathway, which will:
  a. Produce better people living with dementia or Parkinson’s and carer experience of care.
  b. Contribute to workforce satisfaction.
3. All these benefits will produce better value for money in the systems.

These hypotheses were based on some key assumptions.

Assumptions:

1. The health and care systems in which each site works have the requisite enabling mechanisms in place at macro (policy), meso (organisation) and micro (individual) levels.
2. The PROCare4Life solution does not change the care pathway but provides the infrastructure creating opportunities for greater integration.
3. In line with the scope of the ‘usual’ care pathways, primary and community care is not included in the PROCare4Life solution.

By efficiency, it is understood “how well a health care system uses its resources (input) to improve population health (outcome)”, following the definition by the Expert Group on Health Systems Performance Assessment (HSPA), ^35^ measured as subjective perception of results.

### Research techniques

Several complementary research techniques have been used to respond to each of the above questions. The first source of information was the previous data already generated by PROCare4Life consortium members. Surveys and interviews were translated from English into the local languages of the pilot sites: Spanish, Italian, Romanian, German, Portuguese. Before launching them, they were tested with people representing the target groups to assure its suitability for their respective profiles and cultural adaptation. Fieldwork involved 5,127 total participants. With the support of the respective WP leaders and the six pilot sites, the relevant quantitative and qualitative evaluation results ^36^ coming from previous or parallel evaluations done under PROCare4Life implementation period, particularly those gathered through Pilot 3, have been identified and reanalysed.

The second source of information has derived from tailored internal qualitative research among the PROCare4Life consortium partners leading the pilots. Respondents were PROCare4Life team members. A specific interview guide was created, circulated, and responded to in written form by pilot site representatives, focused on gathering comparable information from each pilot site on the integration of the PROCare4Life solution into their existing care pathways, the effects of PROCare4Life, the system’s value for money and the replicability of the model.

Third, additional qualitative information was gathered using personal semi-structured interviews among researchers participating in the pilots. A specific interview guide was created, focused on asking the pilot sites about the specific effects of PROCare4Life on people living with Parkinson’s, dementia and/or comorbidities, their carers, and their healthcare professionals. Key informants were selected at each pilot site to complete these personal interviews, having signed a previous informed consent form. Interviews were held online using Microsoft Teams. The interviews focused on further clarifying the nature and scope of the care services that conformed to their existing care pathways, how the PROCare4Life solution merged into their current care pathways, and whether and how it brought about any service delivery changes. Six 45-minute online interviews were conducted over April and May 2023. In line with the Rapid Evaluation methodology ^37^, handwritten notes were taken right after the interviews and the information was discussed and reviewed by two research team members as close to the time of the interview as possible, to validate the quality and appropriateness of the information gathered.

The fourth source of information for the validation research was created and circulated among technical partners developing the ProCare4Life system. A devoted questionnaire to gather the technical requirements for the future scalability of the PROCare4Life system from the technical point of view was tailored, circulated, and responded to in writing by the technical coordinator and the technical leader of the recognition of user activities, including specific disease related symptoms.

Finally, the analysis of the micro, meso, and macro conditions for scalability of PROCare4Life involved assessing the integrated care best practice assessment using the Scirocco tool for the six pilot sites. The SCIROCCO tool ^38^ was developed with EU funding, aiming to assess the readiness of regions for scaling up integrated care. Scirocco’s results have allowed for further reflection on PROCare4Life’s prospective orientation and, thus, on the scalability of PROCare4Life’s contribution to the integration of care.

Data analysis has involved assessing the consistency of results coming from different sources, thus allowing triangulation of the results. The information has been selected, analysed, and reported according to each of the research questions and validation objectives, detailed in each subsection where the information came from. Quantitative analysis has been performed using SPSS. Qualitative content analysis information has been reported quoting the original responses of the participants, whenever possible, as the structure of the questionnaire is suitable for structured comparison of responses according to key concepts.

## Results

In this section, summaries of the PROCare4Life care model validation results are presented, organised according to the research questions advanced in the methods subsection above. The first subsection below focuses on assessing how the PROCare4Life solution fitted into the existing care pathway in the pilot sites. The second subsection focuses on the effects of PROCare4Life on care performance within the following dimensions: Patients’ experience of care; Equity of access; Patients’ quality of life; Caregivers’ quality of life. The third section focuses on the efficiency of PROCare4Life from the perspective of the healthcare professionals that tested the system. The fourth section focuses on the value for money analysis, operationalised as the frequency of adverse events and avoidable admissions to hospital. Finally, Scirocco best practice responses, which identify a meso-level current level of maturity for future scaling up of PROCare4Life system for each pilot site, are presented.

### How has the PROCare4Life solution ‘fitted’ into the existing care pathway in the pilot sites?

Over the project implementation, a PROCare4Life integrated care pathway was identified, based on the current care provision pathways of each organisation and the additional integrated care features to be advanced by the PROCare4Life system. Development of the project care pathway also took into consideration the stakeholder needs assessed at the beginning of the project, led by Asociación Parkinson Madrid. By the end of pilot 3, and thus of the iterative testing of the PROCare4Life solution, the pilot sites were asked how PROCare4Life actually fitted into their current pathways. One of the pilot sites stated that PROCare4Life’s “solution is fit for most people living with chronic neurological disorders”, being the citizens with mild or moderate forms of cognitive impairment and those with mild or moderate forms of Parkinson’s, the ones that could profit more from the system. In fact, it was considered that the “system was well tolerated and used with enthusiasm by people living with dementia or Parkinson’s and their caregivers, the need for such a solution being high up on their list of needs.” Additionally, it was suggested that “integrating such a solution much earlier in their disease journey would improve and facilitate the use of technology.” ^39^

Another pilot site described how their services must meet the challenge of bringing their organisation closer to people who must confront “distance to our facilities, lack of mobility, dependence and/or disability.” Thus, “PROCare4Life fits as it is a remote support and monitoring system. Its sensors allow assessment of the status of people living with dementia or Parkinson’s on-demand, evaluating eventual changes due to disease evolution and/or modification in pharmacological/rehabilitative treatment. It covers the need for information and communication interventions between people living with dementia or Parkinson’s and professionals through functionalities such as the app/web chat. Also, periodic information reports from the system to caregivers’ emails and allows coordination in delivering services multi-specialized from different departments who use the website. On the other hand, playing cognitive games in this way could be included in the Cognipark therapeutic sessions (cognitive stimulation + occupational therapy) that are given weekly at the association.” ^40^ From the perspective of a third pilot site, the “concept of PROCare4Life would facilitate the communication between all involved in the care of neurological people living with dementia or Parkinson’s and promote the integration of care”. ^41^

The information gathered through the semi-structured interviews with key informants from the six pilot sites confirmed these initial positive findings. When asked if any changes would be needed to be made in their care pathways to accommodate the PROCare4Life solution, the responses of the pilot sites indicated that “Different and adapted versions for people living with dementia or Parkinson’s with severe forms of neurodegenerative disorders (such as dementia, Parkinson’s disease, parkinsonism syndromes, etc.) are needed. Few people living with dementia or Parkinson’s with advanced diseases can understand and use modern technology (even if they are users of modern smartphones). This group of people would require a simpler solution (e.g., just one smart bracelet that doesn’t require human-technology interaction)” ^42^. Another pilot site considered that “The system needs to answer the needs of the care pathway, not the opposite” and that thus it is not still mature enough to be used in clinical routine, although with an interesting and promising concept.” ^43^

However, despite this identified need to further adapt the PROCare4Life system to the real needs of their users, one of the pilot sites declared that “No major changes were necessary to accommodate the PROCare4Life solution”. Not to be missed was the importance of personalized training for the future users, with respondents noting that from their experience: “The most important aspect was to train health and social care professionals in the operation of the system to be carried out by the people living with dementia or Parkinson’s and in the PROCare4Life website through which the citizens living with dementia or Parkinson’s could be monitored”. ^44^

Also, to be considered for future implementation of the system, the double challenge for carers to integrate the PROCare4Life system into their daily routines for the home scenario, while on the organisational level the solution would need to be implemented in the facility for the day care centre or rehabilitation room scenarios. ^45^

### Effects of PROCare4Life on care performance

In this subsection we address the effects of PROCare4Life on the care performance on: people living with dementia or Parkinson’s experience of care; social and gender equity; people living with dementia or Parkinson’s quality of life; and carers’ quality of life. There is one devoted subsection for each one of the previous dimensions of validation.

As a general conclusion, it was confirmed with high consistency between questionnaires and personal interviews, that pilot representatives considered the PROCare4Life idea and underlying model of care very innovative and promising, and believed that it can certainly improve care practice through more enhanced integrated care. One of the pilot sites indicated that: “After going through several improvements, PROCare4Life positively impacted the care of patients with neurodegenerative disorders” ^46^, confirming that: “In general, patients who have used the PROCare4Life system have shown satisfaction with it. They felt that their health was better monitored through the devices, as they had healthcare professionals looking at the information received. In addition, they were aware of the benefits of using technology to collect objective data on the evolution of their symptoms on an ongoing basis, not just through a few medical sessions per year. Special attention to serious symptoms, especially in the area of prevention or to solve problems that arise (e.g., falls).”

Additionally, “In the cases of patients whose caregiver was also involved, there was an increased sense of security, knowing that the caregiver would be regularly informed about the evolution of the patient’s health”. ^47^ However, one of the pilot sites reported not having identified any effect on patients’ experience of care (understood through its 3 main dimensions, personalisation, coordination and continuity), being their target population people living with dementia. ^48^ Another pilot site considered that the identified positive effect might potentially be connected with the increased attention received as a result of participating in the pilot (daycare centre scenario). ^49^

There was no actual implementation of the system in the current care pathways. This fact is consistent with the prototype status of the technology developed, being most of its components around Technology Readiness Level (TRL) 7. The assessment of the changes in the model of care will have to be left for future research to develop further PROCare4Life system.

***People living with Parkinson’s or dementia’s experience of care.*** The results presented in this sub-section are based on some qualitative results from the questionnaires and the analysis of several quantitative databases generated by Pilot 3 research. The different databases have been explored and analysed from the perspective of this article’s objectives, assessing the consistency of their results with the qualitative responses to the questionnaires gathered among pilot site representatives.

In Pilot 3, the Patient Assessment of Chronic Illness Care (PACIC+) was passed to the intervention group, the control group and the replicability study with people living with comorbidities. In total, 26 different questions were posed to participants to understand the quality of the care that they received. These aggregated responses are an adequate assessment for people living with Parkinson’s or Alzheimer’s regarding how much their respective systems have advanced towards people-centred, integrated care, creating a baseline for future interventions from the point of view of their users. However, it should be noted that this questionnaire did not address the specific effects of the PROCare4Life system, but the current quality of care status of their own care provision systems, as some of its questions are closely connected with integrated care. Gender analysis was not feasible for this group of variables due to lack of disaggregated information.

For the PACIC+ questionnaire, the code goes from 1, which means almost never, to 5, meaning always. It allows the identification of a baseline on the perception of the quality of care as experienced by patients. It might be worthwhile to repeat these questionnaires once the PROCare4Life system is further developed and eventually requiring implementation change management, in the future.

**Figure 3.**
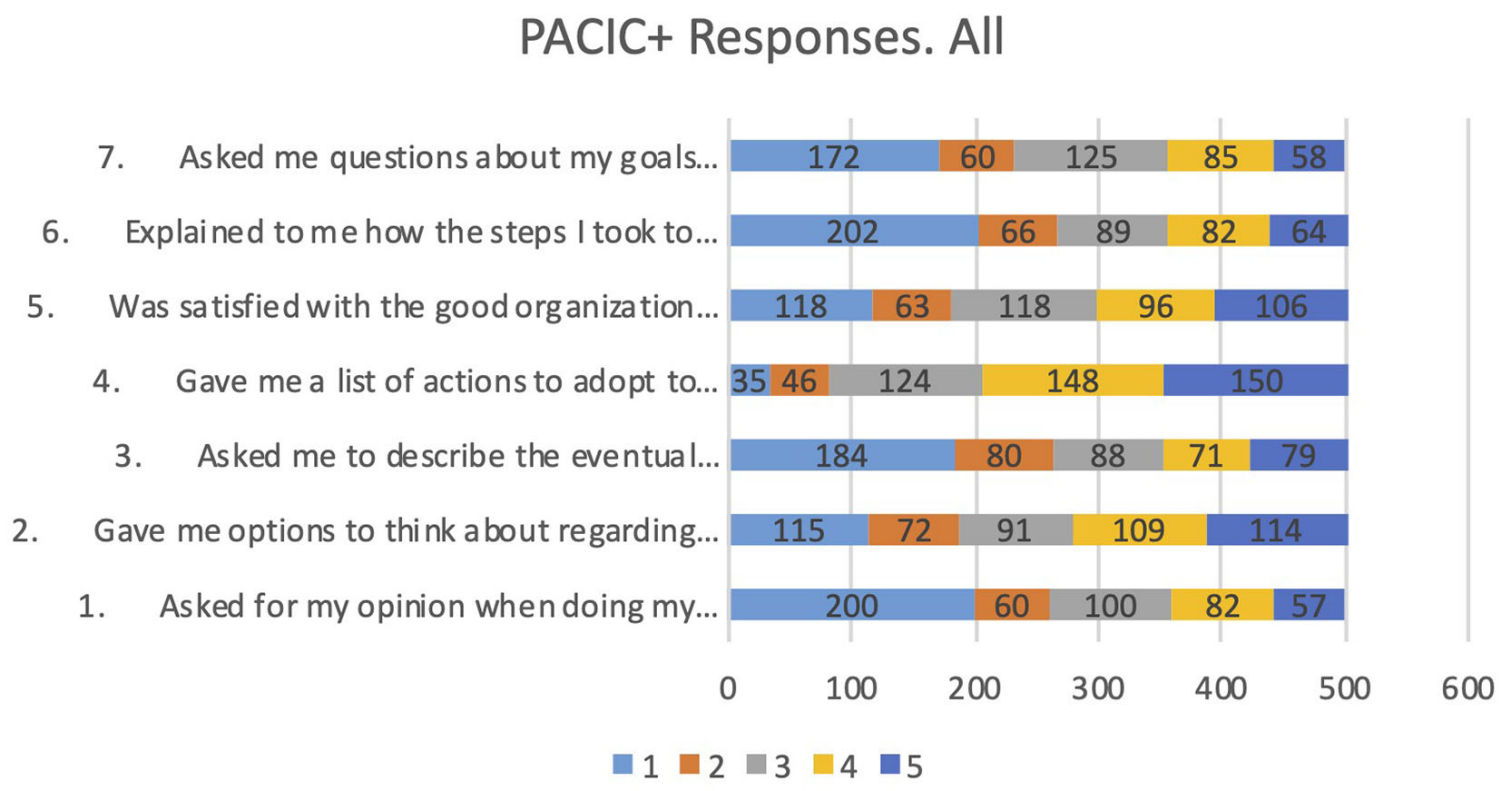
Responses to first 7 questions PACIC+.

The general introduction to the questionnaire is: “We would like to know what kind of help you received by the healthcare team to deal with your illness. This can include your family doctor, nurse, or medical assistant”. The questions included in PACIC+ which are more closely connected with integration of care are those intended to assess how the healthcare professionals supporting the person living with chronic conditions listen, respect them, and have their respective care plan agreed to. Another dimension of the analysis is how much the multidisciplinary coordination, and thus the required continuity, is advanced in their current healthcare services provision. The first seven PACIC+ questions have been included in the analysis, depicted in Figure 3.

The responses to the first seven questions show that the most selected response is “Never” for being asked for their opinion when doing my treatment plan (question 1); Being given options to think about regarding their treatment plan (question 2); Being asked to describe the eventual problems with the medications or their side effects (question 3); Being explained how the steps I took to take care of my illness influenced my progress (question 6); Being asked questions about their goals related to the management of the illness (question 7). More positive results were received regarding being given a list of actions to adopt to improve their health (question 4) or their satisfaction with the good organization of the care provided, that had varied opinions including positive and negative ones (question 5).

No relevant differences have been identified in the responses according to condition, or according to having been control group or intervention group. The responses from PACIC+ show that the quality of care received by chronic patients has room for improvement from a person-centred care.

Further analyses were performed about the primary outcome (quality-of-life) subscores, reported in a subsection below. No significant differences were found regarding the PACIC+ scores.

***Social and gender equity.*** Improving healthcare service access, particularly for vulnerable populations like the ones PROCare4Life is targeting, is closely connected with equity with respect to the healthcare services. ^50^ Equity refers particularly to the equality of outcomes among different population groups. In this subsection the assessment results of how PROCare4Life could be used by people coming from different social backgrounds are addressed. Also, gender differences have been found to affect equity through the PROCare4Life iterative testing.

Regarding the educational levels of the subjects, 68% (n = 361) of the subjects had an education level equal or higher than the secondary level, which is in line with the last data from European Commission (“In 2022, 81.9 % of people aged 25–54 years in the EU had attained at least an upper secondary level of education, compared with 68.4 % of those aged 55–74 years”) ( https://ec.europa.eu/eurostat/statistics-explained/index.php?title=Educational_attainment_statistics#Development_of_educational_attainment_levels_over_time .)

As for the category “environment” (institutionalised vs home based), the intervention was tested in different environments, as follows: home, rehabilitation setting and daycare centers, However, we did not find any significant differences among the three scenarios, except for a lower usability score of the system in daycare centers: in fact, we believe that patients with cognitive decline may face more challenges in navigating the use of the system. In more details, it was found a statistically significant difference in usability of using the system in the daycare center compared with using it at home ( **p = 0.003**) or in the rehabilitation center **(p = 0.006**). Our interpretation of this difference is based on the diagnosis and disease’s severity of patients in the daycare center. The majority were patients with dementia and with significant cognitive impairment, which may interfere with the ability to interact with the PROCare4Life system.

Regarding the mobility of the subjects, at least in the subjects with Parkinson’s disease, we restricted the eligibility criteria as follows: Hoehn and Yahr stages between II-IV, meaning that we excluded bedridden patients.

Finally, on differentiating between early onset vs late onset effect: as the study was focused on older adults (inclusion criteria 65 years old or older) and the mean disease duration was of 8.3 ± 5.7 years, we did not have the chance to explore this aspect neither for Parkinson’s disease (early onset before 50 years old) nor for Alzheimer’s patients (early onset before the age of 65).

In general terms, no relevant social determinant to using and accepting the PROCare4Life system were identified. One of the pilot site representatives has highlighted that people included in the pilots “belonged to all social groups (lower-class, middle-class, and high-class) with all types of educational backgrounds” ^51^, thus being different profiles of citizens helping to co-design the PROCare4Life system, being proven suitable for usage for people coming from different social groups. Another pilot site confronted the challenge of supporting people that had been recently diagnosed with Parkinson’s, and that might be going through their acceptance process in anger or denial; thus “A system such as PROCare4Life solves some of these issues by allowing remote monitoring of the disease and some behaviours, as well as making communication between the people living with dementia or Parkinson’s, the caregiver and their relatives with health and social care professionals accessible”. ^52^ Still, another pilot site considered that the effect on equity of access was none, among their participants living with dementia, because the system is still not suitable for “being considered a valuable tool to use in disease management”. ^53^

Important gender differences have been identified though, particularly regarding reduced social support for women and the over representation of women among caregivers. From the gender perspective, it has been ensured that the participation in the pilots has assured an adequate balance of both males and females: 54% of the participants were males. When considering the two main diagnoses supported by PROCare4Life, a higher presence of men among participants living with Parkinson´s and a higher presence of women among participants living with Alzheimer’s or other similar dementia should be considered when interpreting the project results.

**Figure 4.**
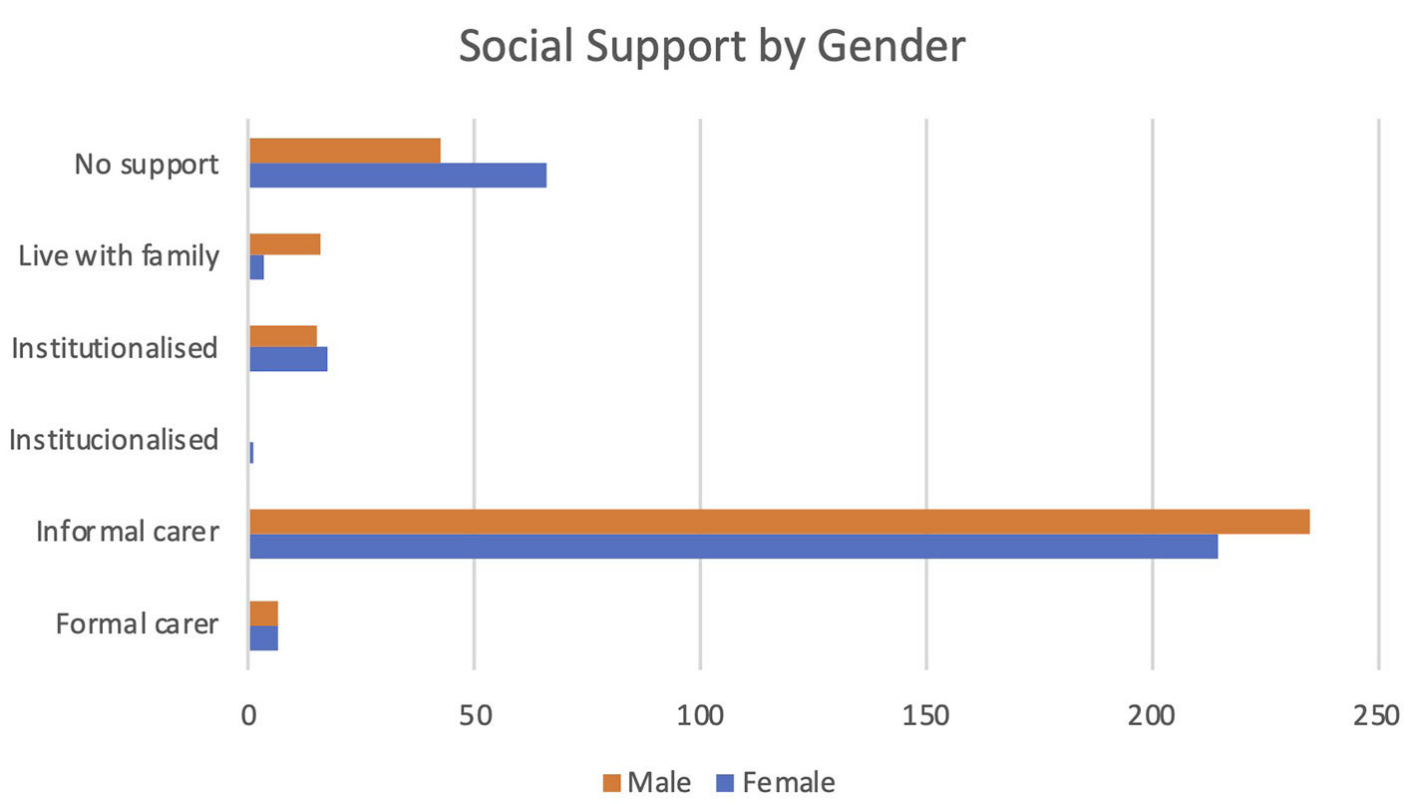
Social support of PROCare4Life participants by gender.

In terms of social support, as can be seen in Figure 4, there are more women with no social support, while there are more men living with their families or enjoying the support of an informal carer. However, figures are quite close for participants institutionalized or enjoying a formal carer for both genders. ^54^ This is an important and very worrying finding about equity in the care burden between men and women, that nevertheless confirms the already known gender disparities in care burden.

**Figure 5.**
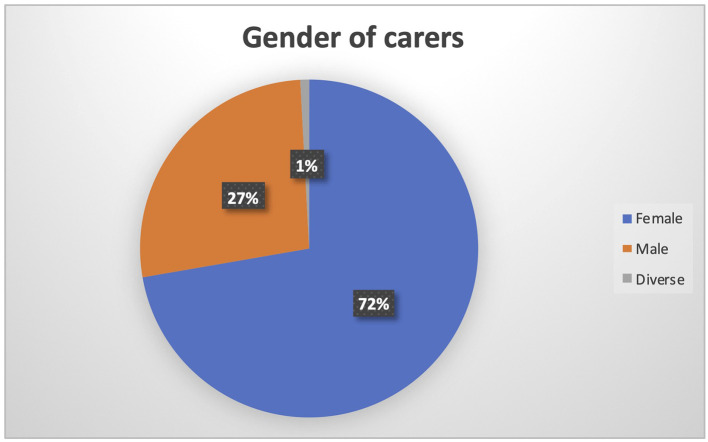
Gender of PROCare4Life Pilot 3 carers.

When considering the gender of carers, it is once more evident the higher burden of women in the care process (N being 126). On the one hand, women represent 72% of the carers, as depicted in Figure 5.

**Figure 6.**
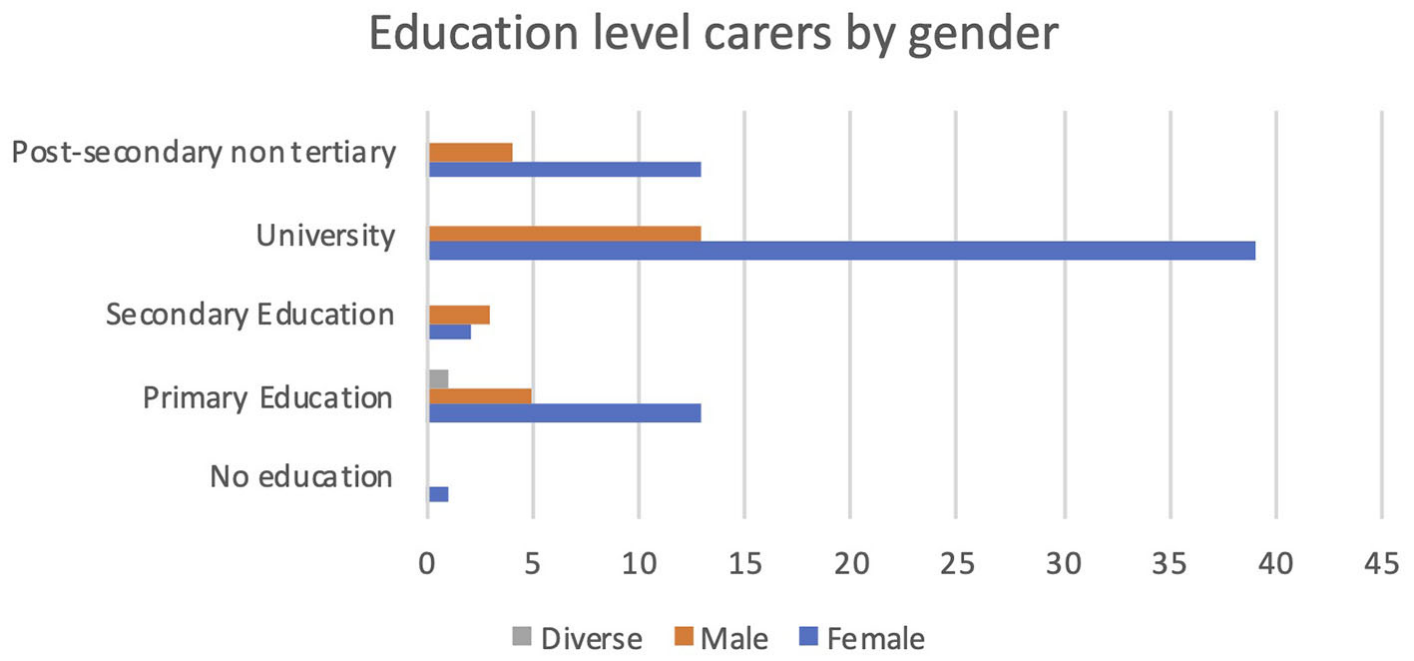
Education level of carers.

Additionally, female carers were much more qualified than the male carers participating in PROCare4Life Pilot 3. The largest groups of carers were females with a university degree. In all educational levels, women were more numerous than men, except for the secondary education level (see Figure 6).

However, it must be noted that these results cannot be attributed to the PROCare4Life system, but to widespread gender social determinants. ^55^ Future developments of the PROCare4Life system should consider these facts to make sure that the system is adapted to the differential needs according to gender in aspects such as training materials or personalised recommendations.

Other aspects such as equality of access and results will require further investigation for the future implementation of PROCare4Life, particularly the adaptation of the system for vulnerable populations, people living in remote areas, among other key targeted populations whose integration is to be the focus of the future development of the platform, according to the EU guidelines.

***People living with Parkinson’s or dementia’s quality of life.*** This is one of the key aspects of the analysis of the PROCare4Life integrated care model validation. The increase in the quality of life of its end users was one of the key objectives of the project. The results have been positive, as the consortium was able to identify a subjective increase in the quality of life of people using it, that most likely relates to the enhanced communication with their care ecosystem, particularly their healthcare professionals. Also highlighted by people living with dementia or Parkinson’s that the constant monitoring made them feel more assured. One of the pilot sites explained it as follows: “In general, patients are aware that health monitoring can help them better understand their health status, allowing them to take preventative measures and adherence to their treatments. Furthermore, they trust that systems like PROCare4Life can detect health problems before they become more serious conditions, which could reduce their risk of complications and improve their health outcomes. Also, monitoring systems that can provide feedback to patients increase their motivation and engagement in health care, can help them feel more empowered in their health care.” ^56^

It has been nevertheless highlighted by other pilot sites that the explanation for the improvement of the quality of life of people living with dementia or Parkinson’s might be linked to other factors responsible for these results, mainly that the healthcare professionals were seen by participants more often and their wellbeing was frequently verified both by their healthcare professionals and carers. ^57^ Again, there was no improvement in the quality of life among some of the participants, people living with dementia in advance stages.

The previously presented qualitative results are congruent with those identified through the EUROQoL questionnaire. The quality of life of people living with dementia or Parkinson’s has been measured with EUROQoL, a standardised questionnaire. Based on 644 cases, some positive results on the quality of life of patients using the system could be identified. However, it should be noted that, due to the error level of 4% for a confidence level of 95%, some of these identified changes could be explained by statistical error. EUROQoL asks the participants to assess their own general health under a 100 points scale, and their mobility, self-care, usual activities, level of pain or discomfort and anxiety or depression under a five-point scale.

**Figure 7.**
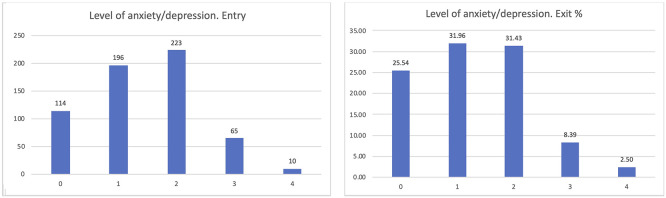
EUROQoL anxiety entry and exit interview.

The most interesting dimension linked to our goals is the level of anxiety of the participants ( Figure 7). PROCare4Life is expected to be able to reduce the level of anxiety or depression of their users, thanks to the reinforced health monitoring, direct communication with their care ecosystem (carers, healthcare professionals) and personalised recommendations. After participating for between 2 and 3 months in pilot 3, people living with dementia or Parkinson’s s confirmed that their levels of anxiety or depression improved after their participation in pilot 3, despite the advance of their chronic conditions. The best possible result for the perceived level of anxiety is zero, representing participants who were feeling good, which has increased from 18.72 to 25.54% from the entry interview to the exit interview, also representing a reduction in the number of those declaring that their anxiety levels were severe or extreme.

**Figure 8.**
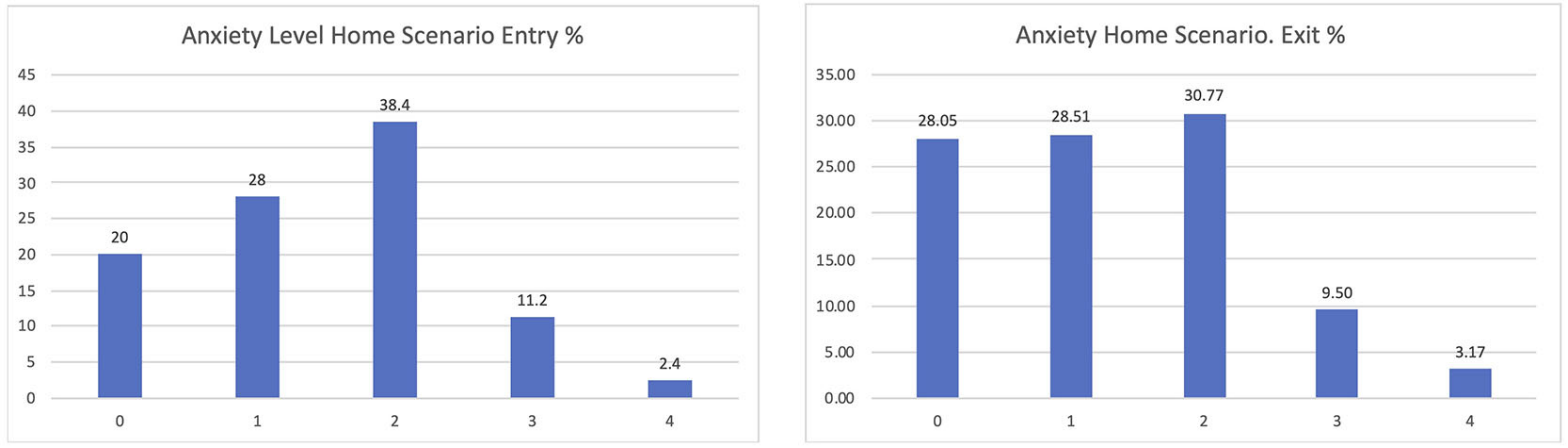
EUROQoL Anxiety levels entry and exit interviews, home scenario.

If considering the different scenarios, these results are further confirmed. Clearer positive effects have been identified in the comparison between the entry and exit interviews for those using the home scenario, passing from 20% declaring zero anxiety to 28.05% when leaving the study ( Figure 8).

**Figure 9.**
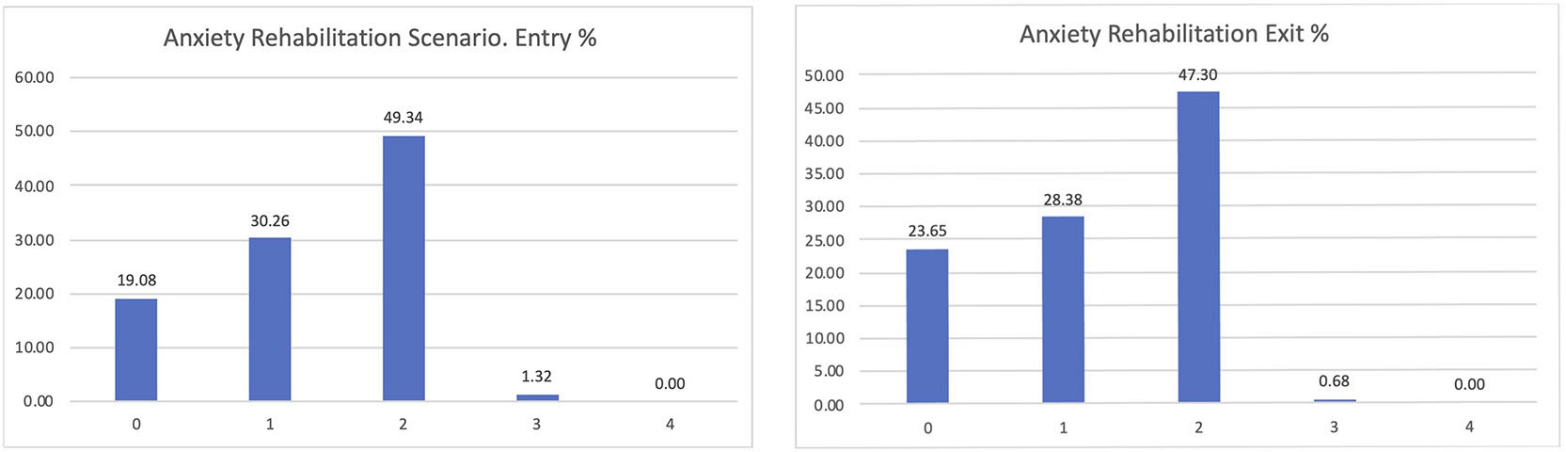
EUROQoL Anxiety levels entry and exit interviews, Rehabilitation scenario.

Similar results were confirmed, with an almost 5% increase in those declaring zero anxiety among the participants in the rehabilitation scenario ( Figure 9).

**Figure 10.**
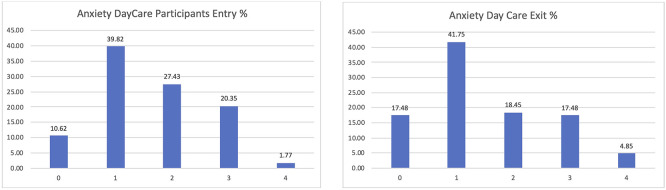
EUROQoL anxiety entry and exit interviews, Day Care scenario.

Among the participants in the Day Care scenario, the lower levels of anxiety were confirmed too, passing from 10 to 17% of those declaring zero anxiety or depression and being reduced to those having previously declared high levels of anxiety or depression ( Figure 10).

No relevant differences were found in the self-perception of their health between entry and exit interviews among participants of Pilot 3, which is coherent with the short period of participation and the natural deterioration of health linked to both chronic conditions, dementia and Parkinson’s. Additionally, it must be noted that it was not a goal of the PROCare4Life project to improve the health of the people being supported by the PROCare4Life system, but to better monitor them, to empower them and to support enhanced communication with their carers and healthcare professionals, to support the future integration of care through multidisciplinary enhanced collaboration and communication. All these changes were expected to improve their quality of life, as shown.

**Figure 11.**
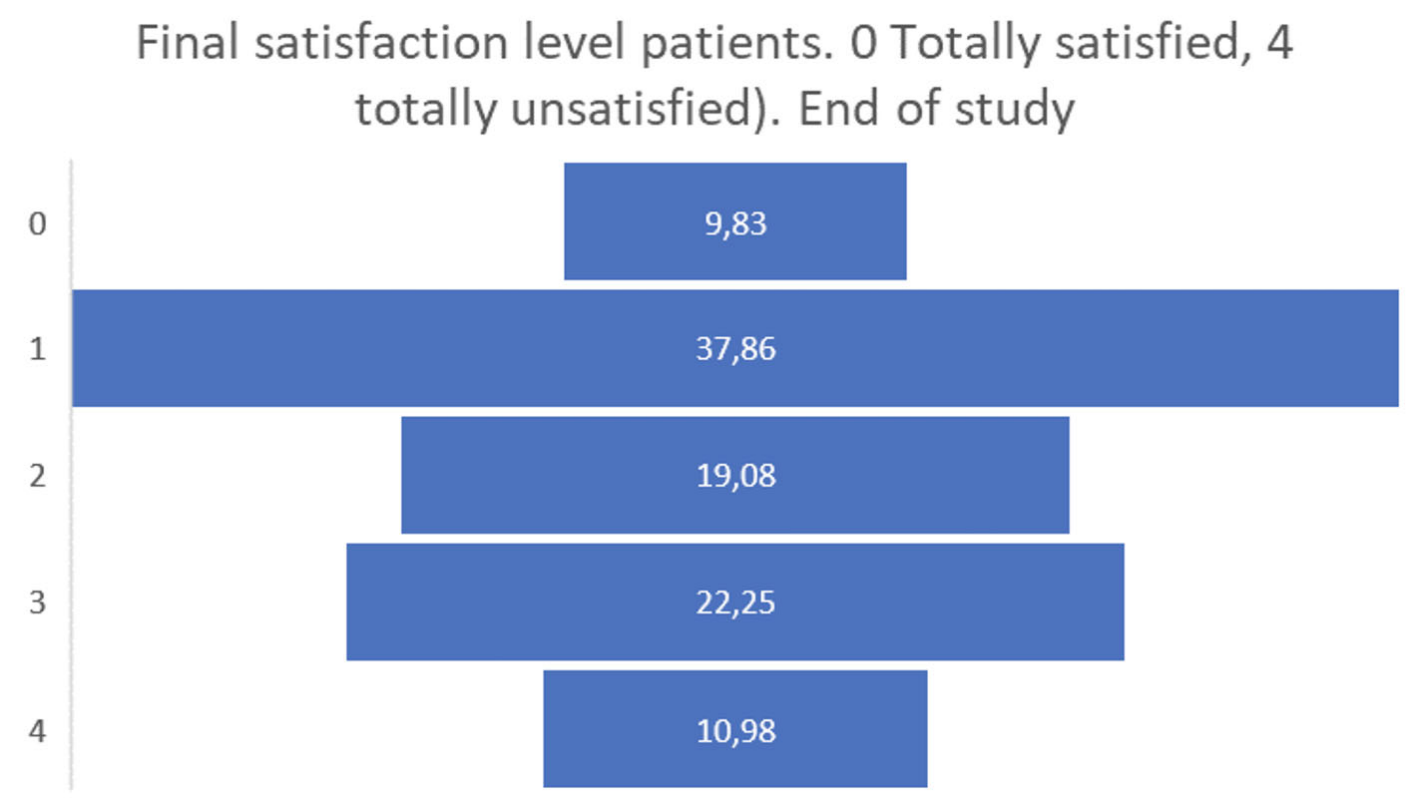
Final satisfaction level participants pilot 3.

Finally, the satisfaction levels of the people living with dementia or Parkinson’s participating in the pilot were overall positive, obtaining a positive assessment of 0 or 1 by almost half of the participants (see Figure 11).

Further analyses were performed about the primary outcome (quality-of-life) subscores. They were performed per group allocation (Experimental Group VS Control Group) and type of system used (Cloud System VS Full System): there was a significant difference in the quality of life for EG VS CG with respect to perceived pain and discomfort (p = 0.03) and anxiety and depression (p = 0.05) (see table below).

**Table 1.** EURO QoL subscores.

	p EG vs CG
**EURO QoL|Pain**	**0.03**
**EURO QoL|Anxiety**	**0.05**
**Assessment of the care received**	
**PACIC+**	0.4

No significant differences were found regarding the PACIC+ scores.

For the future developments of PROCare4Life, it will be key to consider that there has been some positive feedback at the end of the study questionnaires, such as that the people living with dementia or Parkinson’s value highly the activity monitoring and alerts, Fitbit data gathered, the exercise recommendations and its reward system. Also, users very much appreciated the large amount of health data collected and that it was shared among the care ecosystem with carers and healthcare professionals, who can monitor citizens’ health status at any time remotely.

However, it was also made evident that the system was considered complex to use for a relevant number of participants, who complained about having to be reminded to charge the devices, connectivity problems between them, the need to send data by themselves every night or, for some of them, having to wear the Fitbit all the time. Additionally, users repeatedly requested access to their own gathered data and results, since they filled in many questionnaires, but they did not have automatic access to this information. Cognitive games were loved by some but considered too easy or too difficult by others. Depth cameras were not accepted by many end users and the solution was redesigned to use cloud services instead of mini-PCs to make the system simpler, among many other adjustments to respond to the participants’ feedback.

**Figure 12.**
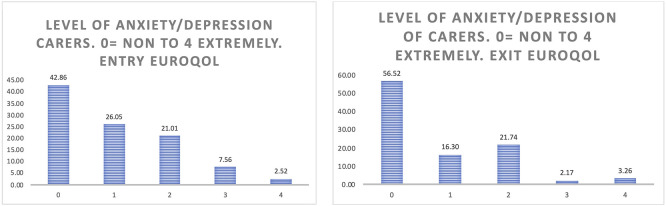
EUROQoL Level of anxiety/depression of carers. Entry and exit interview.

***Carer’s quality of life.*** When considering the anxiety of the carers ( Figure 12), the positive effects of participation in the study are more evident than in the case of people living with Parkinson’s and Alzheimer’s, reflecting more than a 13% increase in those carers who do not feel anxiety or depression at all after having participated in Pilot 3.

**Figure 13.**
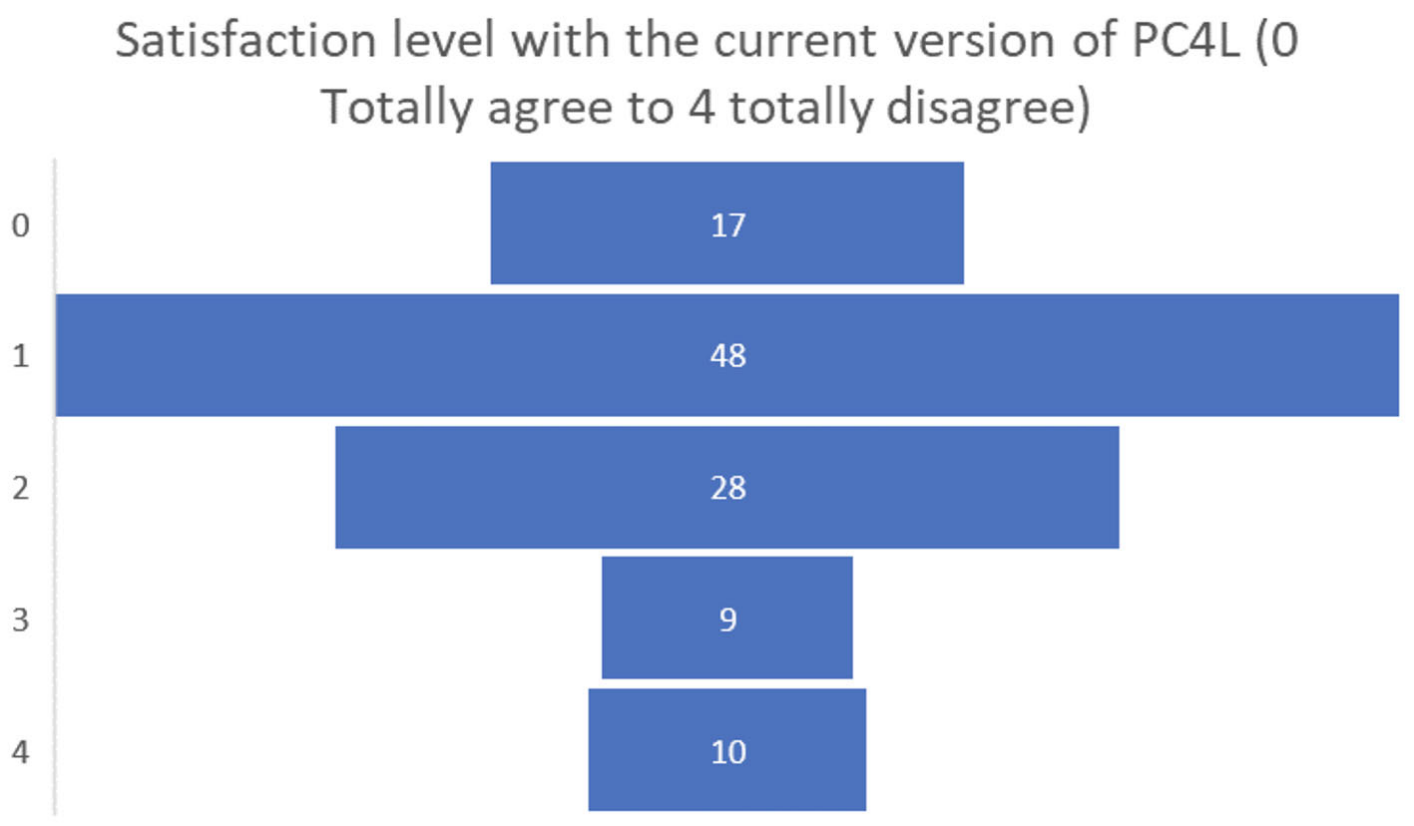
Satisfaction with PROCare4Life system (Carers).

In the end of-study interview completed by carers, it was highlighted that the PROCare4Life system was valuable for them ( Figure 13) because of its continuous monitoring of the people they were taking care of, most usually family members and relatives, highlighting the large amount of health and daily life data gathered by the system. Also highly valued by carers was that PROCare4Life allowed them direct contact with healthcare professionals.

Among the things to be improved in the future, participants consistently highlighted the data transmission issues, the complexity of the platform usage for some of the people living with dementia or Parkinson’s, and the challenging installation and charging of the devices.

Among the final suggestions for future developments of the PROCare4Life system, the most requested have been: the automatization of data collection; increased simplicity and easier to use; less equipment; more diversity and levels of the games, again in clear alignment with the recommendations and requests posed by those they take care of. Their final satisfaction levels were positive, with more than two-thirds of carers considering that their satisfaction level with the current version of PROCare4Life was either total or high.

These results are again largely congruent with those gathered through the questionnaires completed by the pilot sites. In one of the pilot sites, it is considered that the quality of life of the care staff improved, “there is now Wi-Fi in all day care centres and mobile services. And they have been equipped with technical devices. The project participants were temporarily involved by us and needed less care and attention from the care staff during this time”. ^58^ In another pilot site, there is an interesting reflection about cultural determinants of the care activities and care burden and its relationship to PROCare4Life: “Caring for people affected by Parkinson’s disease requires a lot of effort and dedication. The main carers, at some point in the evolution of the disease (more or less prolonged), will suffer in greater or lesser intensity the following symptoms: health problems and physical pains (help in carrying out the basic tasks of daily life); psychological problems including depression and sadness; and social isolation (they devote all their time and effort to care).

Systems like PROCare4Life can provide a sense of security to know that their family member is in a safe environment where they are able to consult doubts with professionals, tranquillity, the feeling of having the situation under control since they are informed periodically about their beloved activities, and specifically notified of any arising risks being aware of patient’s disease evolution.” ^59^

This opinion seems to be also partially shared by the participants of other pilot sites: “The role of the PROCare4Life system in improving the quality of life for patients with neurodegenerative disorders was a subject of conflicting opinions among their carers. In the initial two pilots, most carers faced difficulties installing the system, making them sceptical about its usefulness. However, participants in the third pilot reported that being able to monitor their loved ones’ health significantly improved their quality of life and reduced their anxiety levels”. ^60^ Finally, one of the pilot sites considered that the technical aspects need to be further adapted for dementia participants, not having found any effect on their carers’ quality of life. ^61^

***Healthcare professionals’ efficiency.*** The PROCare4Life system has been designed for health data sharing among patients, carers, and healthcare professionals. This functionality is intended to promote increased multidisciplinary coordination and continuity, given that data sharing is critical for different healthcare professionals to work together on behalf of the patient’s needs and wishes, being a key for integrating care.

**Figure 14.**
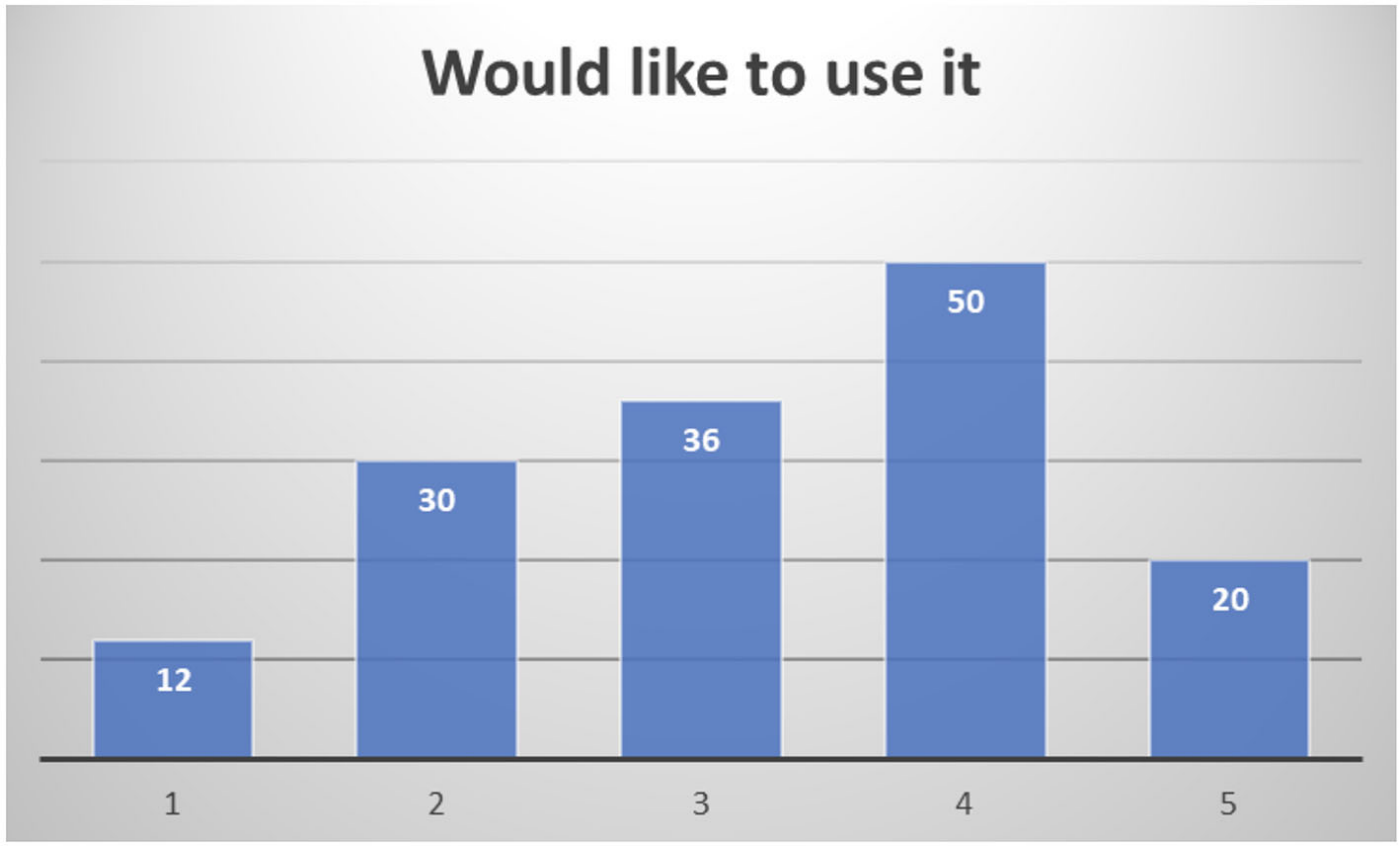
Healthcare professionals' willingness to use PROCare4Life (1 Very Unlikely to 5 Very Likely).

Among the 159 professionals responding to the end-of-study questionnaire, quite different profiles have been included: clinical doctors, nurses, nutritionists, occupational therapists, physiotherapists, psychologists, social workers, speech and language therapists, and also healthcare managers. Their overall assessment of the system is quite positive, considering almost half of the respondents (47.3%) that they would be likely or very likely to use it ( Figure 14).

**Figure 15.**
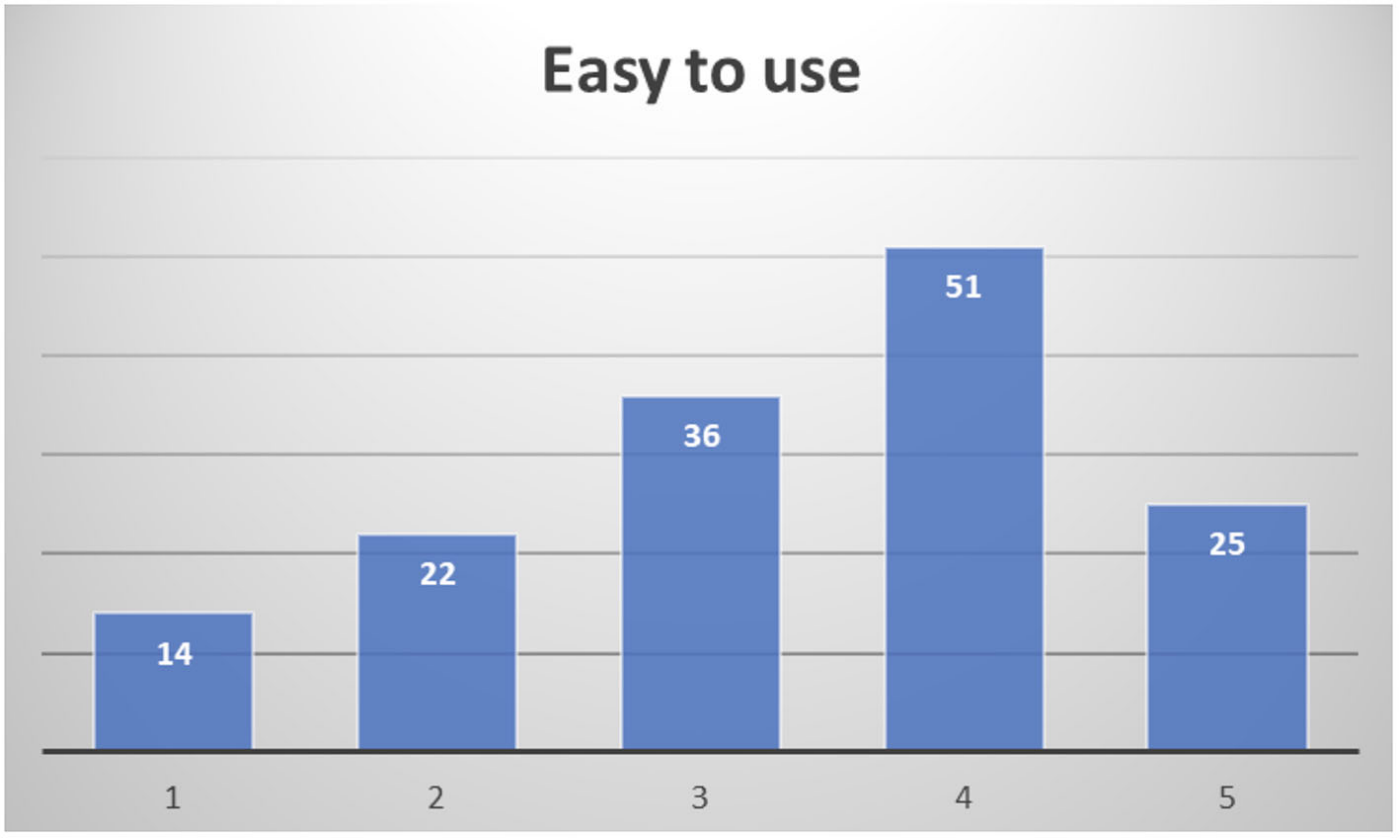
Healthcare professionals' opinion on easiness to use PROCare4Life.

In the final assessment after their participation, about half of the respondents consider it easy to use (51.4%), not expecting to require assistance to use it most of them (28% would consider it likely or very likely to actually require assistance to use it), as depicted in Figure 15.

Among the most valued functionalities of the PROCare4Life system, healthcare professionals who participated in the Pilot 3 showed a great congruence in their responses about the most and least valued, by patients and carers. The most valued is to assist practitioners with data collection, which integrates information for everyone (patient, healthcare professionals, carers), integrates cognitive games that allows direct communication with patients and continuous remote monitoring.

They also considered the system to be a cost-effective way to evaluate patients, easy to navigate and use, that facilitates the work of the healthcare professionals.

Detailed and dynamic monitoring might also be suitable for personalising treatments, thus supporting healthcare professionals with innovative technologies. Again, healthcare professionals detected the same weaknesses identified before when analysing opinions of patients and carers: it was considered a complex system for the patients, time consuming devoted to charging and sending data, app and web app are slow, chat did not always work, information not always appear, difficulties installing and synchronising devices. According to this group of end users, the system needs further adaptation to the target population. These results indicate the lines for future work to refine the prototype.

How about workforce efficiency? Almost 40% of the respondents consider or strongly consider that the system will save time in clinical practice, while another 40.1% consider or strongly consider the opposite, that in fact, it will not save time in clinical practice. A similar distribution of opinions was identified when asking healthcare professionals if they considered the PROCare4Life system cost-effective: 41% agreed or strongly agreed, while 43.8% disagreed or strongly disagreed. Further research might be required to understand the reasons for this distribution of responses.

**Figure 16.**
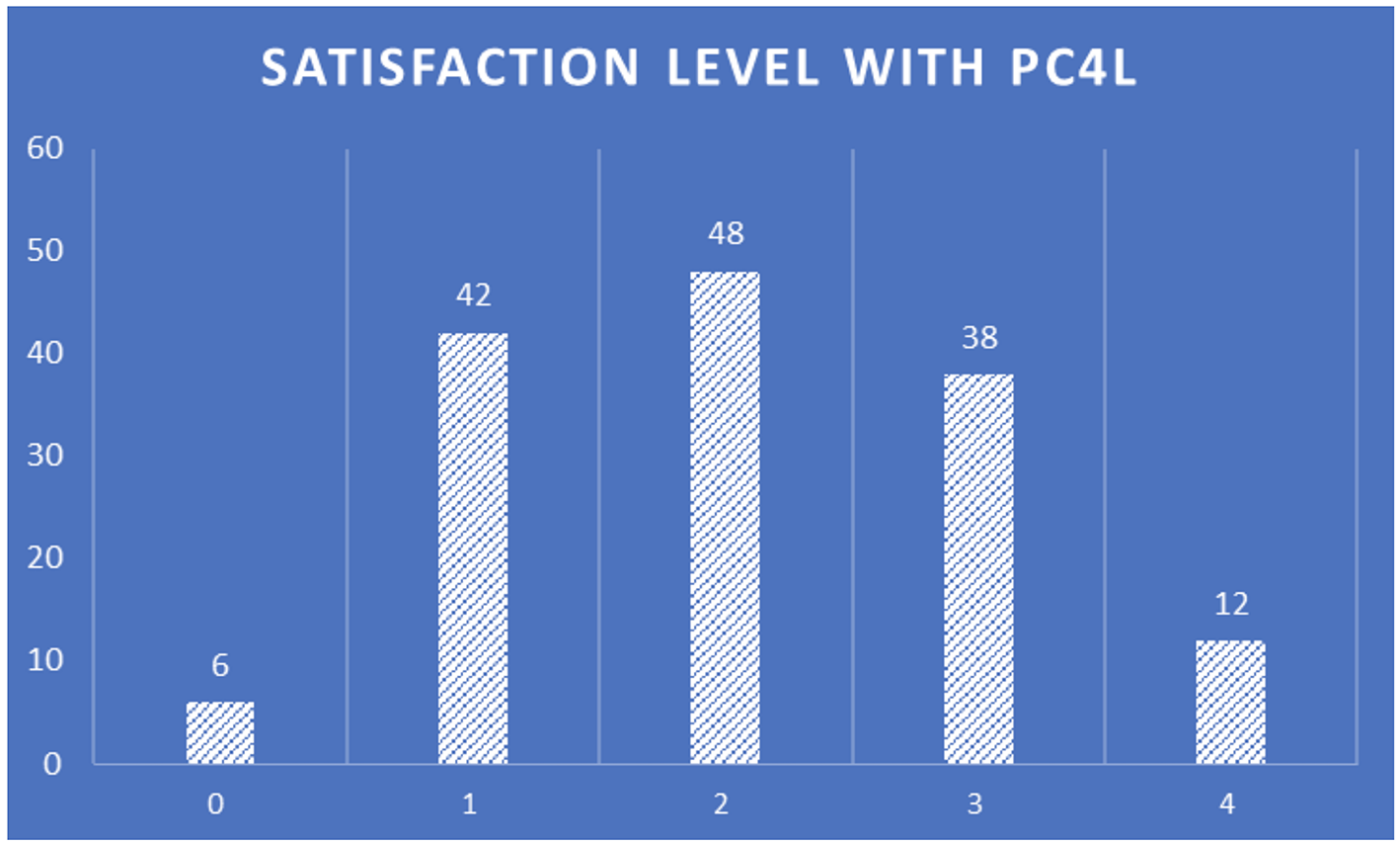
Healthcare professionals satisfaction level with PROCare4Life.

The satisfaction level with PROCare4Life ( Figure 16) has an almost central normal distribution, highlighting the importance of work to adapt the system to increase the satisfaction of healthcare professionals using it in the future versions of the PROCare4Life system, making sure to keep the most valued functionalities and addressing these suggestions of improvement and satisfaction levels.

At this stage of development, the survey for decision-makers reflected that although the system might be able to facilitate the clinical decision-making process (62.5% completely or quite trust this result), almost half of the respondents (49%) do not know if they would pay for it.

However, 69% of the respondents expect that PROCare4Life, thanks to data sharing among different profiles of healthcare professionals, people living with Alzheimer’s, Parkinson’s, or other neurological conditions, will help to improve the healthcare provision towards a more coordinated, continued and people-centred services (the three main characteristics of integrated care healthcare services).

For future development of PROCare4Life, since it is still not mature enough to be brought to the market, ^62^ it must be considered that “implementing an integrated care system can require a significant initial investment in terms of resources and time, so it is important to carefully evaluate costs and long-term benefits”. Additionally, it should be considered that “When an integrated care system improves the care experience of patients, their quality of life, allows access for different social groups, improves the quality of life for caregivers, decreases the frequency of adverse effects and hospital readmissions, and increases worker efficiency, this translates into increased system profitability in several ways: *Cost reduction:* The decrease in the frequency of adverse effects and hospital readmissions means a decrease in medical care costs. In addition, improved worker efficiency can also reduce administrative and personnel costs; *Increased patient satisfaction:* Improved care experience and quality of life for patients and caregivers can result in increased satisfaction and loyalty to the health system, which can attract more patients and increase long-term profitability; *Reputation and credibility improvement:* Successful implementation of an integrated care system can improve the reputation and credibility of the health system and attract more patients and medical care providers; *Workload reduction and increased productivity*: Improved worker efficiency can reduce workload and increase productivity, which can translate into increased long-term profitability”. ^63^

### Results in earlier intervention and management of common symptoms of PD and AD: reducing adverse events and avoidable admissions to hospital

For the PROCare4Life system to be fully integrated in future healthcare provision organisations, it is key to understand its effect on adverse effect and number of admissions to hospital. On these dimensions, evidence is limited and not completely aligned. From the perception of one of the pilot sites, their participants had fewer admissions to hospital. However, they highlighted that the reasons might be “multifactorial, as they felt safer knowing their health was under continuous monitoring” ^64^. However, the other pilot sites could not confirm any positive correlation in this respect.

Several tentative analyses have been performed on the available quantitative databases to identify potential positive effects derived from earlier intervention and management of common symptoms of people living with Parkinson’s and people living with dementia. The expected results were based on the expected reduction of anxiety that could lead to a reduction in the request for non-programmed medical services, most usually emergency services, which are costly services currently offered by healthcare provider organisations.

To validate these expected results, some questions were included in the PROCare4Life interviews: “How has PROCare4Life pilots affected the demand for non-programmed medical resources?”. There are also several clinical symptoms that have been measured at the entry and exit interviews of each one of the participants: number of falls, number of choking events, number of festination events, and number of wandering episodes, whose goal was to contrast the declared health-related symptoms with the information gathered by the PROCare4Life system.

When looking at the raw data, it can be noticed that 84,5% of the participants did not request any non-programmed medical services already when entering the pilot. When exiting it was 88.2% overall. Among those requesting them, there seems to be an apparent global reduction of the total number of participants requesting at least one emergency service after using the PROCare4Life, being reduced from 75 to 45 participants who declared having requested non-programmed medical services before and after entering the PROCare4Life pilots (dropouts not included in the analysis). When comparing the request of non-programmed healthcare services between dementia patients and those living with Parkinson’s or parkinsonism, some differences arise. The difference in requesting one service among people living with dementia dropped 50%, while it dropped 60% among people living with Parkinson’s. Those demanding at least two services dropped 37,5%, These results should be taken cautiously since the number of requested non-programmed services might be based on a longer period than the time spent participating in PROCare4Life pilot 3. Thus, the comparison would not be adequate from the longitudinal perspective. Additionally, no significant differential effect has been identified when comparing the intervention with the control group.

### Macro, meso and micro level results: SCIROCCO integrated care self-assessment

It has been reported different dimensions of the PROCare4Life integrated care model validation, covering subjective and objective, qualitative and quantitative indicators. This last section focuses on the integrated care framework where organisations implanting the pilots have contributed to developing the PROCare4Life system. The SCIROCCO integrated care self-assessment tool has provided an opportunity to collect the perception of the respondent on the maturity of the different domains presented. It was a subjective exercise of reflection about the maturity of the components that would enable the creation of integrated care pathways or models. Moreover, the respondents of this exercise were representatives of the pilot sites which do not represent all the stakeholders involved in the care of people with neurodegenerative conditions in their correspondent areas. For this reason, the results of this exercise should not be interpreted and do not represent a consensus about the maturity of the system in the region.

PROCare4life could only be part of fully integrated care pathways and models if there is enough maturity at the macro level. Moving towards integrated care and using technology such as PROCare4Life for such purpose would probably require changes at the macro level, not only at the micro (service) and meso (organisational) levels. To explore to what extent it would be possible to move PROCare4Life system integration of care forward in each country, PROCare4Life pilot representatives were requested to use the SCIROCCO tool able to identify how mature their own health and care systems are. As explained in the methodology section, the SCIROCCO self-assessment tool is an online self- assessment tool with the objective of assessing a region’s readiness for integrated care along 12 dimensions ^65^. Table 2 provides the scores for each dimension and each one of the pilot sites. To capture the maturity level, Scirocco identifies the responses on a scale from 1 to 6 in all the 12 dimensions, being feasible to represent the information with all the dimensions in a table, included above.

**Table 2.** Summary of Scirocco responses from the six pilots.

	**UHB**	**UMF**	**CNS**	**WBW**	**APM**	**CCPP**	**Mode**	**Median**
Q1 - Readiness to Change	2	2	1	3	3	3	3	2.5
Q2 - Structure & Governance	2	2	3	2	3	3	2	2.5
Q3 - Digital infrastructure	4	3	1	2	3	3	3	3
Q4 - Process Coordination	4	3	2	4	3	2	4	3
Q5 - Funding	2	1	0	2	3	3	2	2
Q6 - Removal of inhibitors	1	1	0	2	3	2	1	1.5
Q7 - Population Approach	4	1	3	3	3	3	3	3
Q8 - Citizen Empowerment	4	1	2	4	2	3	4	2.5
Q9 - Evaluation Methods	2	3	0	4	3	4	3	3
Q10 - Breadth of Ambition	1	1	0	3	3	2	1	1.5
Q11 - Innovation Management	1	2	1	3	3	4	1	2.5
Q12 - Capacity Building	4	3	0	3	2	3	3	3

The responses of the SCIROCCO self-assessment show a remarkably high variation in the perception of the maturity of different domains among different pilot sites. The highest variations are observed in the digital infrastructure, population approach, citizen empowerment, evaluation, innovation management and capacity building. On the other hand, there is a consensus showing the lowest maturity level on the funding, removal of inhibitors and breath of ambition domains. The responses of different pilot sites agree to a certain level on the following elements that could limit the implementation of PROCare4Life in an integrated care pathway or models for neurodegenerative conditions: system-wide innovation; structure and governance; digital infrastructure; workforce capacity and capability; accessibility; patient and public engagement.

There is still room for preparing and increasing the maturity of the pilot sites health and care systems to effectively advance integrated care and maximise the use of technology and support solutions such as PROCare4Life.

## Discussion

Some limitations of the study need to be considered. First, the short duration of the pilot studies (2-3 months) limits the ability to assess long-term impacts, such as sustained quality of life improvements or reduction in healthcare utilization like hospital admissions. Second, the exclusion of individuals with severe forms of dementia or Parkinson’s raises questions about the system’s inclusivity and applicability to the most vulnerable populations. Simplified versions of the system could address this gap. Third, the quality of life may be partially attributable to increased contact with healthcare professionals during the pilot. Fourth, the results are largely based on qualitative results, requiring further quantitative research in areas such as cost-effectiveness and reduced healthcare utilization. The PROCare4Life platform is an ambitious, complex effort, and we have seen how some of the main challenges we faced throughout the implementation and validation of the platform were either unforeseen or somewhat underestimated. ^66^ One of the main ones would be the difficulty in aligning user needs and what the solution can offer. PROCare4Life involved an order of thousands of users, in five different countries and three different scenarios. At the end of the project, it cannot be said with 100% certainty that there are no open questions. The system still needs to continue being improved and iteratively tested with real users since the final Technology Readiness Level (TRL) is still not market ready. Despite TTL-7 achieved, usability challenges and feedback indicate the need for further iterations to meet end-user needs fully.

One of the areas that have proved to be challenging when looking at the implementation of PROCare4Life from a technical perspective has to do with the target population. The users of the platform are older adults living with dementia or Parkinson’s affected by neurodegenerative issues in various degrees. The system’s complexity emerged as a common critique. Participants struggled with device synchronization, data transmission, and the need for manual actions, undermining the goal of seamless usability, particularly for elderly users. In the future, it would be beneficial for IT experts working in the platform to be aware of the limitations that this puts in the operation of the different components of the platform, and most particularly, in the user devices. Simplicity and automation of the solution have been advanced by the development team, in response to the demands from users. Therefore, IT experts with background or joint expertise with human-machine interfaces, quality of experience, or some kind of medical background would be at an advantage in understanding the challenges in their development of components in the platform. Further research to understand the relationship between efficacy of implementation and co-design of digital tools with people living with dementia or Parkinson’s disease and their careers will inform this enhancement. Equally, further research should explore the relationship between technology such as the PROCare4Life solution on the transformation of care pathways to be more inclusive with people actively involved in clinical and care decision-making.

PROCare4Life technology has been codesigned and iteratively tested through the three pilots with the support of real people, representatives of its future users. Some positive results linked to improved satisfaction of people living with dementia or Parkinson’s, carers, and partially healthcare professionals, have been identified. The increased support for multidisciplinary, people-centred care has been advanced through data sharing and communication between people living with dementia or Parkinson’s, their carers and healthcare professionals. Gender equity remains a challenge that will need to be further addressed, considering the differences in social support and caregiver role identified. A considerable percentage of caregivers were females with a high level of qualification and thus expected to have advanced digital skills. Also, the percentage of female people living with dementia or Parkinson’s and not having personal caregivers to be further taken into account for future developments of the platform.

Acknowledging the existing barriers to system integration into existing care pathways, future research will be required for developing concrete strategies for scaling and integrating PROCare4Life systems. Further integration of care using PROCare4Life solution will require a solid change management strategy including aspects such as organisational strategy, tasks, people, data, and technological needs. ^67^ Next steps in the future implementation of PROCare4Life should bridge the gap between theory and practice of integrated care initiatives, making sure to include the analysis of the power dynamics involved in integrated care change management for healthcare professionals involved, ^68^ together with their respective perspectives ^69^ and needs. The costs and effects the integration ^70^ of the PROCare4Life system will also need to be further researched, together with the analysis of the system on non-programmed care services demand. Further research should focus to enhance our understanding of scaling digital solutions like PROCare4Life to advance the integration of care in the future.

## Ethical considerations

All the consortium partners have signed a data protection agreement, being the documents’ this article is based upon classified as confidential by PROCare4Life Grant Agreement.

PROCare4Life large scale pilots for the iterative testing and codesign of the ICT solution obtained ethical approvals from the respective local Ethical Committees of each pilot site as follows.

(1) Wohlfahrtswerk für Baden-Württemberg: Approval from the Ethical commission of the University of Münster for the User-Requirements Study (2020-37-MB), Pilot 1 (2020-59-MB-FA), Pilot 2 (2021-15-MB-FA2), Pilot 3 (2022-29-MB-FA4)

(2) Asociación Parkinson Madrid: Approval from the Ethical commission of Hospital Clínico San Carlos for the User-Requirements Study (20/453-E) Pilot 1 (20/656-E) Pilot 2 (21/220-E) Pilot 3 – Clinical Study (22/392-E)

(3) Casa di Cura Policlinico: Approval from Comitato Etico Milano Area 2 of the Fondazione IRCCS Ca’ Granda Ospedale Maggiore Policlinico for the User-Requirement Study (ID Sperimentazioni 1796) Pilot 1 (OSMANI-20/10/2020-0034210-U). Pilot 2 OSMAMI-26/07/2021-0032326-U Pilot 3 (OSMANI-22/09/2022-0044110-u)

(4) Campus Neurologico Senior: Approval of the Comissao de Ética Campus Neurologico Senior (BIO72685263) Pilot 1 (N. Ref. 13-20) Pilot 2 (N. Ref. 3-2021) Pilot 3 (N. Ref. 6-2022-R)

(5,6) UHB and UMF, both located in Romania: Comisia de Etica e Cercatarii of the Spitalul Clinic Colentina. Pilot 1 (Nr. 25/30.10.2020) Pilot 2 (Nr. 24/28.09.2021) Pilot 3 (Nr. 7/19.07.2022)

## Data Availability

PROCare4Life consortium is under the obligation to protect results because of a legitimate interest of confidentiality under Grant Agreement number 87522. While data is not published openly, the request should be sent by email to Claudia Louro, clouro@kinetikoshealth.com Joao with the following information: - Name: [Requestor’s Name] - Position: [Requestor’s Position] - Area of Study: [Requestor’s Area of Study] - Institution: [Requestor’s Institution] - Email: [Requestor’s Email]

## Footnotes

^1^
“Estimating the Prevalence of Dementia in Europe.” n.d. https://www.alzheimer-europe.org/sites/default/files/alzheimer_europe_dementia_in_europe_yearbook_2019.pdf.

^2^
See European Federation of Pharmaceutical Industries and Associations (EFPIA), Microsoft Word - EFPIA AD Platform - Health System Readiness_20200911_FINAL.docx, 20 September 2023.

^3^
“Let’s Talk about Parkinson’s.” n.d. https://www.age-platform.eu/sites/default/files/EPDA-Political_Manifesto_Parkinson.pdf.

^4^
Maresova P, Klimova B, Novotny M, Kuca K. Alzheimer’s and Parkinson’s Diseases: Expected Economic Impact on Europe-A Call for a Uniform European Strategy. J Alzheimers Dis. 2016 Oct 4;54(3):1123-1133. doi: 10.3233/JAD-160484. PMID: 27567862.

^5^
Alberto Belmonte-Hernández, Yusuf Can Semerci, João Pedro Proença, and Sergio Romera-Giner. 2022. PROCare4Life: An integrated care platform to improve the quality of life of Parkinson’s and Alzheimer’s patient. In The 15th International Conference on PErvasive Technologies Related to Assistive Environments (PETRA’22), June 29-July 1, 2022, Corfu, Greece. ACM, New York, NY, USA, 7 pages. https://doi.org/10.1145/3529190.3534727

^6^
Tedeschi, M *et al.* 2019 Digital health initiatives and the policy context in European integrated health systems. International Journal of Integrated Care, 19(S1): A546, pp. 1–8, DOI: dx.doi.org/10.5334/ijic.s3546

^7^
Primary health care: making our commitments happen. Realizing the potential of primary health care: lessons learned from the COVID-19 pandemic and implications for future directions in the WHO European Region. Copenhagen: WHO Regional Office for Europe; 2022. Copenhagen: WHO Regional Office for Europe; 2022. Licence: CC BY-NC-SA 3.0 IGO.

^8^
Lennox-Chhugani, N. A User-Centred Design Approach to Integrated Information Systems – A Perspective. International Journal of Integrated Care, 2018; 18(2): 15, 1–4. DOI: https://doi.org/10.5334/ijic.4182

^9^
Lennox-Chhugani N 2021 Artificial intelligence as a driver of shifting power towards patients – How could new technology enable integrated person-centered care? International Journal of Integrated Care 22(S1) A1, pp. 1–8, DOI: doi.org/10.5334/ijic.ICIC21013

^10^
“The European Innovation Partnership on Active and Healthy Ageing (EIP on AHA)|Shaping Europe’s Digital Future.” n.d. Digital-Strategy.ec.europa.eu. https://digital-strategy.ec.europa.eu/en/policies/eip-aha .

^11^
“Get Involved! – In4aha.” 2020. Tehnopol.ee. 2020. https://innovation4ageing.tehnopol.ee/contact-us/.

^12^
Henderson, Donna; Pavlickova, Andrea; Lewis, Leo. 2016 Scalability and transferability of good practices in Europe: What does it take? International Journal of Integrated Care, 16(6):A191, pp. 1–8, DOI: http://doi.org/10.5334/ijic.2739

^13^
Darley A, Carroll Á. Conducting Co-Design with Older People in a Digital Setting: Methodological Reflections and Recommendations. *International Journal of Integrated Care,* 2022; 22(4): 18, 1–11. DOI: https://doi.org/10.5334/ijic.6546

^14^
Project funded under the same Topic Call, see Project ValueCare - Value-based methodology for integrated care supported by ICT, 8 September 2023.

^15^
Care, Integrated. 2019. “VIGOUR - Scaling up Integrated Care.” Vigour-Integratedcare.eu. 2019. https://www.vigour-integratedcare.eu/.

^16^
“Home - InCareHeart.” 2024. InCareHeart. June 5, 2024. https://incareheart.eu/.

^17^
CareMatrix. 2023. “Home - Carematrix.” Carematrix. October 3, 2023. https://carematrix.eu/.

^18^
“Users and Citizens - ECare.” 2022. ECare. 2022. https://ecare-pcp.eu/users-and-citizens/.

^19^
“European Project ROSIA - European Union’s Horizon 2020 - ROSIA-PCP.” 2023. ROSIA-PCP. August 7, 2023. https://rosia-pcp.eu/project-rosia/.

^20^
“Home.” 2017. VCare Project. January 4, 2017. https://vcare-project.eu/.

^21^
“IDEA-FAST.” 2019. Idea-Fast.eu. 2019. https://idea-fast.eu/.

^22^
“Tender Health – TeNDER Project.” 2019. Tender-Health.eu. April 8, 2019. https://www.tender-health.eu/.

^23^
Lennox-Chhugani, Niamh. 2018. “A User-Centred Design Approach to Integrated Information Systems – A Perspective”, in *International Journal for Integrated Care (IJIC),* Volume: 18, Page/Article: 15, doi: 10.5334/ijic.4182.

^24^
Carroll, A. Twomey, Valerie. White, Barry. 2018 Identifying barriers to Implementation of New Care Models, a Multi-Stakeholder Engagement Study. International Journal of Integrated Care, 18(S2): A48, pp. 1-8, DOI: dx.doi.org/10.5334/ijic.s2048

^25^
Øvretveit, J 2017 Digital Technologies Supporting Person-Centered Integrated Care – A Perspective. International Journal of Integrated Care, 17(4): 6, pp. 1–4, DOI: https://doi.org/10.5334/ijic.3051

^26^
Diller, Stephanie. Hannah, Aiden. 2022. “Integrated Care Systems: achieving an integrated future through shared data”, in *Thoughts for the Centre* of Deloitte Centre for Health Solutions.

^27^
Stein KV, Goodwin N, Aldasoro E, Miller R. The Integrated Care Workforce: What does it Need? Who does it Take? *International Journal of Integrated Care,* 2023; 23(3): 1, 1–3. DOI: https://doi.org/10.5334/ijic.7686

^28^
Ferri Sanz M, Vallina Acha B, Ferrando García M. Co-Design for People-Centred Care Digital Solutions: A Literature Review. *International Journal of Integrated Care,* 2021; 21(2): 16, 1–17. DOI: https://doi.org/10.5334/ijic.5573

^29^
Steele Gray C. Integrated Care’s New Protagonist: The Expanding Role of Digital Health. *International Journal of Integrated Care,* 2021; 21(4): 1, 1–5. DOI: https://doi.org/10.5334/ijic.6437

^30^
Macadam, Margaret. 2008. *Frameworks of Integrated Care for the Elderly: a Systematic Review. CPRN Research Report.* Ontario: Ministry of Health and Long-Term Care.

^31^
PROCare4Life codesign and testing was implemented at the home scenario in all the pilot sites. Regarding the other scenarios, PROCare4Life pilot 3 was implemented as follows: CNS: in the inpatient setting, at the Rehabilitation Centre; CCI: in the inpatient setting, at the Gym; UMF: at the Day Hospital; UHB: in the rehabilitation room at the hospital; APM: at the Rehabilitation Room; WBW: at the Day Care Centre room.

^32^
Lennox-Chhugani, Niamh. 2021. “Integrated Care. Defining for the Future through the Eye of the Beholder”, in *International Journal of Integrated Care*, Vol. 21, Page/article 13, https://doi.org/10.5334/ijic.6427

^33^
Bartlett, R., Gjernes, T., Lotherington, A.-T. and Obstefelder, A. (2018), Gender, citizenship and dementia care: a scoping review of studies to inform policy and future research. Health Soc Care Community, 26: 14–26. https://doi.org/10.1111/hsc.12340

^34^
“Demographics|Parkinson’s Foundation.” n.d. www.parkinson.org. https://www.parkinson.org/advancing-research/our-research/parkinsons-outcomes-project/demographics.

^35^
Safety, Food. n.d. “TOOLS and METHODOLOGIES to ASSESS the EFFICIENCY of HEALTH CARE SERVICES in EUROPE an Overview of Current Approaches and Opportunities for Improvement Report by the Expert Group on Health System Performance Assessment.” https://health.ec.europa.eu/system/files/2020-03/2019_efficiency_en_0.pdf.

^36^
Ahmed M, Marín M, Bouça-Machado R, How D, Judica E, Tropea P, Bentlage E, Brach M. Investigating Users’ and Other Stakeholders’ Needs in the Development of a Personalized Integrated Care Platform (PROCare4Life) for Older People with Dementia or Parkinson Disease: Protocol for a Mixed Methods Study. JMIR Res Protoc. 2021 Jan 12;10(1):e22463. doi: 10.2196/22463. PMID: 33433394; PMCID: PMC7837994.

^37^
Chisnall G. (2023) An Introduction to Rapid Evaluation, LSHTM Center for Evaluation https://www.lshtm.ac.uk/newsevents/news/2023/introduction-rapid-evaluation; Vindrola-Padros C. *et al*. (2021) Rapid, Responsive and Relevant? A systematic Review of Rapid Evaluations in Health Care, *American Journal of Evaluation*, vol. 42 (1), 13–27.

^38^
“SCIROCCO – Scirocco – Scaling Integrated Care in Context.” 2018. Scirocco-Project.eu. 2018. https://www.scirocco-project.eu/.

^39^
Questionnaire responses 1

^40^
Questionnaire responses 2

^41^
Questionnaire responses 3

^42^
Questionnaire responses 1

^43^
Questionnaire responses 3

^44^
Questionnaire responses 2

^45^
Questionnaire responses 4

^46^
Questionnaire responses 1

^47^
Questionnaire responses 2

^48^
Questionnaire responses 3

^49^
Questionnaire responses 4

^50^
World Health Organization. 2024. “Improving Service Access and Quality.” www.who.int. 2024. https://www.who.int/activities/improving-service-access-and-quality.

^51^
Questionnaire responses 1

^52^
Questionnaire responses 2

^53^
Questionnaire responses 3

^54^
Non-binary or other gender identities were considered, although the gender distribution did not allow for further differential analysis of other categories.

^55^

European Institute for Gender Equality. (2021). *Gender inequalities in care and consequences for the labour market.* European Institute for Gender Equality (EIGE) Cascella Carbó GF, García-Orellán R. Burden and Gender inequalities around Informal Care. Invest Educ Enferm. 2020 Feb;38(1):e10. doi: 10.17533/udea.iee.v38n1e10. PMID: 32124578; PMCID: PMC7871478. Alonso, C., Brussevich, M., Dabla-Norris, E., Kinoshita, Y., & Kochhar, K. (2019). Reducing and Redistributing Unpaid Work: Stronger Policies to Support Gender Equality. *IMF Working Paper*, *19*(225). https://doi.org/10.2139/ssrn.3482305

^56^
Questionnaire responses 2

^57^
Questionnaire responses 1 and 4

^58^
Questionnaire responses 4

^59^
Questionnaire responses 2

^60^
Questionnaire responses 1

^61^
Questionnaire responses 3

^62^
Questionnaire responses 3 and 4

^63^
Questionnaire responses 2

^64^
Questionnaire responses 1

^65^
“Maturity Model in Practice – Scirocco Assessment Tool – SCIROCCO.” n.d. https://www.scirocco-project.eu/maturity-model-in-practice-scirocco-assessment-tool/.

^66^
Gangas P, Judica E, Marin M *et al.* PROCare4Life lessons learned [version 2; peer review: 2 approved, 1 approved with reservations]. Open Res Europe 2024, 3:147 ( https://doi.org/10.12688/openreseurope.16304.2)

^67^
Thomas H. Davenport and Thomas C. Redman. 2020. “Technology And Analytics. Digital Transformation Comes Down to Talent in 4 Key Areas”, in *Harvard Business Review,* Digital Transformation Comes Down to Talent in 4 Key Areas (hbr.org), 22 September 2023.

^68^
Lennox-Chhugani N. InterDisciplinary Work in the Context of Integrated Care – a Theoretical and Methodological Framework. *International Journal of Integrated Care,* 2023; 23(2): 29, 1–11. DOI: https://doi.org/10.5334/ijic.7544

^69^
See an example of analysis from the point of view of Directors and Governors of the NHS, 1. Dixon A, Storey J, Rosete AA. Accountability of Foundation Trusts in the English NHS: Views of Directors and Governors. Journal of Health Services Research & Policy. 2010;15(2):82–89. doi: 10.1258/jhsrp.2009.009078

^70^
Rocks S, Berntson D, Gil-Salmerón A, Kadu M, Ehrenberg N, Stein V, Tsiachristas A. Cost and effects of integrated care: a systematic literature review and meta-analysis. Eur J Health Econ. 2020 Nov; 21(8):1211-1221. doi: 10.1007/s10198-020-01217-5. Epub 2020 Jul 6. PMID: 32632820; PMCID: PMC7561551.

