# Supplementary material for: PROCare4Life integrated care model validation
Source: Open Res Eur. 2026 Apr 16;4:3. Originally published 2024 Jan 3. [Version 3] doi: 10.12688/openreseurope.16678.3 (PMC13213360; doi:10.12688/openreseurope.16678.3)
Supplement: Peer review history

# Reviewer report: Reviewer response for version 3

**DOI:** 10.21956/openreseurope.25555.r73432

**Published:** 2026-05-26

**Authors:** Hein Van Hout

**Affiliations:** 1 Vrije Universiteit Amsterdam, Amsterdam, The Netherlands

It reports a series of studies developing and testing a new tech solution integrating multimodal assessments that should inform and alert patients, informal carers, profesionals. This is an ambitious but complex and challenging endeavor.

 After reading the paper it is still not clear to me which patient and carer needs are addressed by the tech solution. Some examples would be helpful.

 It is still not clear to me what the solution exactly entails. Multiple modes collect data. What type of outcomes / alerts are shared with end users? What is displayed on the dashoards? With what frequency? Some examples would be helpful.

 The methods and results are well structured. The figures and tables, however, could be more informative by adding an explanatory legenda of the answering categories. The sentences in Figure 3 , PACIC+ , are not readable. Multiple figures show frequencies where propotions would make it to easier to grasp the results. What is the EUROQOL Anxiety score? Do they mean the depression or anxiety item? The Scirooco answering options remain unclear.

 In the writing style, sometimes, rather long sentences are used which makes it hard to follow. The discussion section and reflects the findings well and includes limitations and next steps.

If applicable, is the statistical analysis and its interpretation appropriate?

Partly

Is the study design appropriate and is the work technically sound?

Yes

Is the work clearly and accurately presented and does it engage with the current literature?

Partly

Are the conclusions drawn adequately supported by the results?

Yes

Are sufficient details of methods and analysis provided to allow replication by others?

No

Are all the source data and materials underlying the results available?

No

Reviewer Expertise:

Science of care for older persons

I confirm that I have read this submission and believe that I have an appropriate level of expertise to confirm that it is of an acceptable scientific standard, however I have significant reservations, as outlined above.

# Reviewer report: Reviewer response for version 2

**DOI:** 10.21956/openreseurope.19460.r44589

**Published:** 2024-11-27

**Authors:** Liang-Kung Chen

**Affiliations:** 1 National Yang Ming Chiao Tung University, Taipei, Taiwan

The PROCare4Life project, funded by the EU, developed a personalized integrated care solution to support older individuals with dementia, Parkinson’s disease, or comorbidities. The system, tested through three pilot waves in six EU countries, incorporated multidisciplinary code sign, real-time monitoring, and AI-driven insights, aiming to enhance self-management, quality of life, and caregiver support. I read the report with great interest and please find my specific comments as below:

1. The paper ambitiously tackles integrated care for neurological disorders. However, the short duration of pilot studies (2-3 months) limits the ability to assess long-term impacts, such as sustained quality-of-life improvements or reduction in healthcare utilization like hospital admissions.
2. While the TRL-7 status of the PROCare4Life system demonstrates near-market readiness, it is clear that significant refinements are required before real-world implementation. Usability challenges and feedback indicate the need for further iterations to meet end-user needs fully.
3. The exclusion of individuals with severe forms of dementia or Parkinson’s raises questions about the system's inclusivity and applicability to the most vulnerable populations. Simplified versions of the system could address this gap.
4. The system's complexity emerged as a common critique. Participants struggled with device synchronization, data transmission, and the need for manual actions, undermining the goal of seamless usability, particularly for elderly users.
5. The study highlights quality-of-life improvements but admits these may be partially attributable to increased contact with healthcare professionals during the pilot. The lack of a clear distinction between system effects and external influences weakens the study’s conclusions.
6. Despite robust data collection via surveys and monitoring tools, the analysis often relies on qualitative findings. Quantitative outcomes, such as cost-effectiveness and reduced healthcare utilization, are not adequately explored or statistically validated.
7. The study acknowledges the barriers to system integration into existing care pathways, such as organizational inertia and the need for macro-level policy support. Future research should focus on developing concrete strategies for scaling and integrating such systems.

If applicable, is the statistical analysis and its interpretation appropriate?

Yes

Is the study design appropriate and is the work technically sound?

Yes

Is the work clearly and accurately presented and does it engage with the current literature?

Yes

Are the conclusions drawn adequately supported by the results?

Yes

Are sufficient details of methods and analysis provided to allow replication by others?

Yes

Are all the source data and materials underlying the results available?

Yes

Reviewer Expertise:

Frailty, sarcopenia, dementia and health service research

I confirm that I have read this submission and believe that I have an appropriate level of expertise to confirm that it is of an acceptable scientific standard, however I have significant reservations, as outlined above.

# Reviewer report: Reviewer response for version 2

**DOI:** 10.21956/openreseurope.19460.r43905

**Published:** 2024-09-04

**Authors:** Anthony Scerri

**Affiliations:** 1 University of Malta, Msida, Malta

No further comments.

If applicable, is the statistical analysis and its interpretation appropriate?

Not applicable

Is the study design appropriate and is the work technically sound?

Partly

Is the work clearly and accurately presented and does it engage with the current literature?

Partly

Are the conclusions drawn adequately supported by the results?

Partly

Are sufficient details of methods and analysis provided to allow replication by others?

Partly

Are all the source data and materials underlying the results available?

Partly

Reviewer Expertise:

Dementia care

I confirm that I have read this submission and believe that I have an appropriate level of expertise to confirm that it is of an acceptable scientific standard.

# Reviewer report: Reviewer response for version 1

**DOI:** 10.21956/openreseurope.18010.r38743

**Published:** 2024-04-22

**Authors:** Anthony Scerri

**Affiliations:** 1 University of Malta, Msida, Malta

This project developed and evaluated PROCARE4Life aPeRsOnalised Integrated CARE Solution for Elderly facing several short- or long-term conditions and enabling a better quality of Life. This digital tool aims to empower and improve the quality of life of older persons living with Parkinsons' disease, Alzheimer's disease and dementia. The artivle focuses mostly on the evaluation of Pilot study 3 (validation and impact) although reference to the other two pilot studies is given. Whilst the authors attempt to give an overview of the impact of the project, the methods are findings are not clearly described, limiting the reader of have a clear understanding of the implications of the findings. These are some more specific comments that seek improve the writeup of this article:

 Use terms that are language appropriate (e.g. older persons not elderly)

 - Abstract: Explain what is TRL7 when you first refer to it.

 - Pilot 3: Validation and impact of the final version of PROCARE4LIFE system: +1624 – what does the + mean?  -How many participants were recuited?

 - What is the healthcare professionals’ efficiency? – What do you mean by efficiency?

 - Some grammatical errors noted (e.g. The second source of information has been derived from tailored internal qualitative research among…). Consider proof-reading the article. 

 - Briefly explain what the Scirocco tool is:

 -Were the surveys and interview questions translated into the respective languages.  If so, how did you ensure that the questions asked remained consistent in different countries?

 - Explain more clearly who were the participants for all sources of information. Explain in more detail how many participants you had for each source of information.

 - What specific type of qualitative analysis was used e.g. content, thematic etc.

 - With regards to the PACIC+ questionnaire, consider giving some data about the statistical difference between the findings obtained from the intervention group and the control group (e.g. p-values)

 - With regards to the characteristics of the participants in Pilot 3, consider checking whether they represent the population of older persons in the respective countries (e.g. in terms of educational level reached). Moreover, these findings do not give an indication whether the intervention supports particularly categories of persons with Parkinson’s disease or dementia (e.g. early onset vs late onset, mobile vs immobile participants, institutionalised vs home based etc…) Some further analysis on the effectiveness of the intervention for these sub-categories could add to the richness of the study.

 - With reference to the description of the EUROQOL levels of anxiety and depression, consider giving some inferential statistics (i.e. is the difference at entry and exit point statistically significant)?

 - Some explanation in the methodology section is needed of how the project ensured that ethical and GDPR principles were adhered to and maintained throughout the project.

*- “The Mediterranean culture (generalizing) often develops a series of erroneous thoughts about care: high degree of guilt (for thinking that they do not do everything right, for thinking that they do not dedicate enough time or effort to it), care as the sole responsibility of the carer (not shared), with great difficulty in asking for help*”

 With regards to the statement above, is there any evidence of this generalisation? How is this related to the evidence collected and to the outcomes? Consider removing.

 - Explain more clearly what the Scirocco responses mean in Table 1 (e.g. what are the range of values)

If applicable, is the statistical analysis and its interpretation appropriate?

Not applicable

Is the study design appropriate and is the work technically sound?

Partly

Is the work clearly and accurately presented and does it engage with the current literature?

Partly

Are the conclusions drawn adequately supported by the results?

Partly

Are sufficient details of methods and analysis provided to allow replication by others?

Partly

Are all the source data and materials underlying the results available?

Partly

Reviewer Expertise:

Dementia care,

I confirm that I have read this submission and believe that I have an appropriate level of expertise to confirm that it is of an acceptable scientific standard, however I have significant reservations, as outlined above.

# Response

**Published:** 2024-05-31

**Authors:** PILAR GANGAS

**Affiliations:** Scientific Advise Unit, Consejo Superior de Investigaciones Cientificas, Madrid, Spain, Spain

Thank you for your valuable suggestions and recommendations, that we have incorporated into this new version of the article as follows: - What is TRL7 explained in the abstract when first referring to it. 

 - Number of people involved in the pilot corrected to the final figure of 5127 participants in total.

 - Referenced and defined what is understood by the healthcare professionals’ efficiency

 - Article proof read by an English speaking communication professional.

 - Scirocco tool briefly explained.

 - Translation and testing of survey questionnaires described in more detail.

 - Explained more clearly who were the participants.

 - Specified the type of analysis was used.

 - Findings from PACIC+ and EURO-QoL further included and explained.

 - Checking on the characteristics of the participants in Pilot 3 and their representation of older people in the population included.

 - Ethical and GDPR principles further explained.- Removed the sentence on the “Mediterranean culture”

 - Scirocco tool further explained.

 - Contribution from David Linnane reviewing and editing the document acknowledged including him as additional author.
